# Genes and Variants Underlying Human Congenital Lactic Acidosis—From Genetics to Personalized Treatment

**DOI:** 10.3390/jcm8111811

**Published:** 2019-11-01

**Authors:** Irene Bravo-Alonso, Rosa Navarrete, Ana Isabel Vega, Pedro Ruíz-Sala, María Teresa García Silva, Elena Martín-Hernández, Pilar Quijada-Fraile, Amaya Belanger-Quintana, Sinziana Stanescu, María Bueno, Isidro Vitoria, Laura Toledo, María Luz Couce, Inmaculada García-Jiménez, Ricardo Ramos-Ruiz, Miguel Ángel Martín, Lourdes R. Desviat, Magdalena Ugarte, Celia Pérez-Cerdá, Begoña Merinero, Belén Pérez, Pilar Rodríguez-Pombo

**Affiliations:** 1 Centro de Diagnóstico de Enfermedades Moleculares, Centro de Biología Molecular Severo Ochoa, UAM-CSIC, CIBERER, IDIPAZ, 28049 Madrid, Spain; ibravo@cbm.csic.es (I.B.-A.); rnavarrete@cbm.csic.es (R.N.); anaisabel.vega@scsalud.es (A.I.V.); prsala@cbm.csic.es (P.R.-S.); lruiz@cbm.csic.es (L.R.D.); mugarte@cbm.csic.es (M.U.); cpcerda@cbm.csic.es (C.P.-C.); bmerinero@cbmcsic.es (B.M.); 2 Unidad de Enfermedades Mitocondriales y Enfermedades Metabólicas Hereditarias, Hospital Universitario 12 de Octubre, CIBERER, 28041 Madrid, Spain; mgarciasilva@salud.madrid.org (M.T.G.S.); emartinhernandez@salud.madrid.org (E.M.-H.); pilar.quijadaf@salud.madrid.org (P.Q.-F.); 3 Unidad de Enfermedades Metabólicas Congénitas, Hospital Universitario Ramón y Cajal, 28034 Madrid, Spainsinziana.stanescu@salud.madrid.org (S.S.); 4 Dpto. de Pediatría, Hospital Universitario Virgen del Rocío, 28034 Sevilla, Spain; mbuenod@yahoo.es; 5 Unidad de Nutrición y Metabolopatías, Hospital Universitario La Fe, 46026 Valencia, Spain; vitoria_isi@gva.es; 6 Servicio de Neurología Infantil, Complejo Hospitalario Materno Insular, 35016 Las Palmas de Gran Canaria, Spain; mtolbra@gmail.com; 7 Unidad de Enfermedades Metabólicas, Hospital Clínico Universitario de Santiago, IDIS; CIBERER, 15706 Santiago de Compostela, Spain; Maria.Luz.Couce.Pico@sergas.es; 8 Unidad de Enfermedades Metabólicas, Hospital Universitario Miguel Servet, 50009 Zaragoza, Spain; igarciaji@salud.aragon.es; 9 Unidad de Genómica, Parque Científico de Madrid, 28049 Madrid, Spain; ricardo.ramos@fpcm.es; 10 Laboratorio de Enfermedades Mitocondriales y Neuromusculares, Instituto de Investigación del Hospital, de Octubre, CIBERER, 28041 Madrid, Spain; mamcasanueva.imas12@h12o.es

**Keywords:** congenital lactic acidosis, mitochondrial dysfunction, metabolomics datasets, clinical-exome sequencing, RNA analysis, antisense therapy for mitochondrial disorders, healthcare, mitochondrial morphology

## Abstract

Congenital lactic acidosis (CLA) is a rare condition in most instances due to a range of inborn errors of metabolism that result in defective mitochondrial function. Even though the implementation of next generation sequencing has been rapid, the diagnosis rate for this highly heterogeneous allelic condition remains low. The present work reports our group’s experience of using a clinical/biochemical analysis system in conjunction with genetic findings that facilitates the taking of timely clinical decisions with minimum need for invasive procedures. The system’s workflow combines different metabolomics datasets and phenotypic information with the results of clinical exome sequencing and/or RNA analysis. The system’s use detected genetic variants in 64% of a cohort of 39 CLA-patients; these variants, 14 of which were novel, were found in 19 different nuclear and two mitochondrial genes. For patients with variants of unknown significance, the genetic analysis was combined with functional genetic and/or bioenergetics analyses in an attempt to detect pathogenicity. Our results warranted subsequent testing of antisense therapy to rescue the abnormal splicing in cultures of fibroblasts from a patient with a defective *GFM1* gene. The discussed system facilitates the diagnosis of CLA by avoiding the need to use invasive techniques and increase our knowledge of the causes of this condition.

## 1. Introduction

Congenital lactic acidosis (CLA) is a rare condition that is mainly due to a range of inborn errors of metabolism that result in defective mitochondrial function. Lactic acidosis results from the accumulation of lactate and protons in body fluids. A single elevated blood lactate event can have adverse consequences; naturally, sustained hyperlactatemia has an even worse prognosis [1]. CLA is associated with defects in the genes coding for enzymes involved in pyruvate oxidation, the Krebs cycle and gluconeogenesis and is a hallmark of primary mitochondrial disorders (which can involve any of ~1500 mitochondrial or nuclear genes). Since any organ or tissue can be affected by impaired energy production, the associated symptoms and signs of CLA can be very varied and the diagnostic workup is usually complex [2,3,4]. Certainly, the systematic screening of all target organs (heart, muscle, brain, eyes, ear, liver, endocrine system, etc.) must be performed [5], which usually involves biopsies being taken. In addition, the interpretation of the biochemical evidence provided by biomarkers is not always straightforward. Elevated blood and cerebrospinal fluid (CSF) lactate are certainly diagnostic clues that point towards CLA. Alterations in other biomarkers of mitochondrial disorders, such as pyruvate, alanine or acyl-carnitines or cofactors such as free-thiamine or CoQ10 [6,7,8], contribute to address the diagnosis of primary mitochondrial disorders, although are not fully specific and can be detected associated to other secondary mitochondrial dysfunctions. That is the case of CoQ10 levels [8]. The use of a scoring system based on the Consensus of Mitochondrial Disease Criteria (MDC) [9] can help, as can the use of novel computational diagnostic resources such as the Leigh Map [10] but a final diagnosis always requires a genetic analysis be performed.

Next generation sequencing (NGS) has positively influenced diagnosis rates for all heterogeneous genetic disorders. The use of extended gene panels, whole exome sequencing (WES), whole genome sequencing (WGS) and RNA sequencing, has increased diagnostic yield of mitochondrial disorders from 10%–20% in the pre-NGS era to close 50% in the NGS-era [11,12,13,14]. There is now a growing rational for performing sequencing first [15] and treating biochemical analyses as a means of understanding the clinical significance of genetic findings. Indeed, the present work confirms the diagnostic value of combining biochemical profiling and targeted DNA and/or RNA testing to deliver information that minimizes the need for invasive and/or more specialized biochemical tests that delay a diagnosis being reached.

## 2. Experimental Section

### 2.1. Patients

The study subjects were 39 patients (18 males and 21 females, all neonates or infants) who together provided a representative sample of the broad spectrum of clinical signs and symptoms of the patients with suspected CLA referred to our laboratory between 1996 and 2017 (Table S1). All were clinically suspected of having CLA but with different levels of supporting evidence (imaging, biochemical or cellular functional assay results). Most of the patients’ plasma and urine samples were profiled by ion-exchange chromatography, gas-chromatography mass-spectrometry or high-performance liquid chromatography/tandem mass spectrometry, checking for amino acids, urine organic acids and plasma acyl-carnitines and other metabolic studies [16,17]. The results were compared to those for healthy controls but without specific matching for gender or nutritional status. Pyruvate carboxylase and/or pyruvate dehydrogenase activity had already been measured in 23 of the 39 patients (Table S2) but note these results were not used in the present analysis. Histochemical analyses of biopsy materials and enzymatic analyses of mitochondrial respiratory chain complexes activity had not been performed for most patients.

Written informed consent to include the patients in the study was provided by their parents. The study protocol adhered to the Declaration of Helsinki and was approved by the Ethics Committee of Universidad Autónoma de Madrid.

### 2.2. Genetic Analysis

#### 2.2.1. Clinical Exome Sequencing

Genomic DNA was extracted from peripheral blood or fibroblast extracts using the MagnaPure system (Roche Applied Science, Indianapolis, IN, USA) and subjected to massive parallel sequencing using the Illumina^®^ Clinical-Exome Sequencing TruSight™ One Gene Panel (Illumina, San Diego, CA, USA) as previously described [18]. A minimum coverage of 30× was achieved for 95% of the target bases (mean depth of coverage 115×).

#### 2.2.2. Mitochondrial DNA Sequencing

DNA extracted from patient blood samples or skin fibroblasts was checked for large-scale mtDNA rearrangements and mutations according to the Illumina Human mtDNA Genome Kit. VCF files were generated and analysed using Human mtDNA Variant Processor and mtDNA Variant Analyzer software (Illumina, San Diego, CA, USA) (https://blog.basespace.illumina.com/2016/02/25/human-mtdna-analysis-in-basespace/). Sequence variants were annotated according to the MITOMAP database [19]. The mtDNA-server platform (https://mtdna-server.uibk.ac.at/index.html#!pages/home) was used to detect heteroplasmy and to assign mtDNA haplogroups [20]. To detect deletions, the mean coverage for the analysed intervals was calculated and normalized with respect to the mean coverage for all the target intervals. Deleted intervals were then detected by comparing the normalized mean coverage of the test sample with the mean coverage of the control samples.

#### 2.2.3. Variant Prioritization and Pathogenicity Prediction of Nuclear DNA Variants

Candidate variants were filtered to be rare and disruptive to protein function. Variants were considered rare when they appeared with a minor allele frequency (MAF) of <0.5% within the GnomAD database. Variations shared by multiple patients were removed (since CLA is a rare condition it is unlikely that the same variation would be shared by many people). The filtered results only contemplated variants that affected a protein by their coding for a structural variation or their provoking an ablation, deletion, frame-shift, start loss, splice site or stop gain. Filtering also included the presence of gene variants previously associated with each patient’s phenotype and which were annotated in the Human Gene Mutation Database (HGMD, professional version 2019.2) https://portal.biobase-international.com/hgmd/pro/start.php. Although variants inconsistent with a recessive mode of inheritance were initially filtered out, these samples were recovered if the changes were located in genes known to cause congenital lactic acidosis. For missense changes, potential pathogenicity was evaluated using the web platform VarSome (https://varsome.com/) [21]. This brings together data from the dbSNP, ClinVar, gnomAD, RefSeq, Ensembl, dbNSFP, Gerp, Kaviar, CIViC databases and runs the DANN, dbNSFP, FATHMM, MetaLR, MetaSVM, Mutation Assessor, PROVEAN, GERP, LRT and MutationTaster-prediction programs. To complete the analysis of the impact of missense changes on protein structure, function and conservation, the MutPred (http://mutpred1.mutdb.org/) [22] and Panther (http://pantherdb.org/tools/csnpScore.do) [23] prediction programs were also used.

Potential 3’ and 5’ splice sites were analysed as previously described [24] using the default settings of Alamut^®^ Visual Interactive Bio v2.7.1 software. Those variants prioritized to be causal of CLA were confirmed by conventional Sanger sequencing using the BigDye Terminator Cycle Sequencing Kit (Applied Biosystems, Foster City, CA, USA), using both patient genomic DNA and that of the progenitors if available.

The mutation nomenclature employed followed the Human Genome Variations Society Database (HGVS v15.11. format) (http://www.HGVS.org/varnomen/). The DNA variant numbering system was based on the corresponding cDNA sequence, taking nucleotide +1 as the A of the ATG translation initiation codon in the reference sequence.

#### 2.2.4. High-Density Genotyping

A genome-wide scan of 610,000 SNPs was conducted as previously described [25] at the Spanish National Genotyping Centre (CEGEN, www.cegen.org) using the Illumina 610-Quad Beadchip Kit (Illumina, San Diego, CA, USA) according to the manufacturer’s recommendations.

#### 2.2.5. mRNA Studies

500 ng of total RNA were extracted from dermal fibroblasts using the RNeasy Micro kit (Qiagen, Hilden, Germany) and used as a template for reverse transcription PCR (RT-PCR), making use of the NZY First-Strand cDNA synthesis kit (NZYTech, Lisbon, Portugal). PCR amplification was performed using the PCR Supreme NZY Taq II kit (NZYTech, Lisbon, Portugal) with primers designed to amplify the full-length cDNAs according to the cDNA GenBank sequences listed below.

The abundance of full length or aberrant *GFM1* transcripts was evaluated by massive parallel sequencing of cDNA amplicons. Specific amplicons were generated employing primers that included an extended tail (listed in Table S3) and used for library preparation. Libraries were completed by 2-step PCR using the Access Array Barcode Primers for Illumina Sequencers (Fluidigm Corporation, San Francisco, CA), pooled and sequenced in MiSeq (Illumina) in paired-end format of 2 × 300, reaching a depth of >50,000 reads.

### 2.3. Cellular Studies

#### 2.3.1. Cell Culture

Control and patient dermal fibroblasts were grown under standard conditions in minimal essential medium (MEM) containing 1 g/L of glucose supplemented with 2 mmol/L glutamine, 10% foetal bovine serum (FBS) and antibiotics. The cell lines CC2509 (Lonza, Basle, Switzerland), NDHF (PromoCell, Heidelberg, Germany) and GM8680 (Coriell Institute for Medical Research, Camden, NJ, USA) were used as controls. Most experiments were performed when fibroblasts were at 80% confluence.

#### 2.3.2. CoQ10 Measurement

Total CoQ10 was measured by liquid chromatography/tandem mass spectrometry (LC/MS/MS), using CoQ9 as an internal standard, in extracts obtained from two 100 mm plates (P100) of fibroblasts grown under standard conditions. Pelleted cells were resuspended in 125 µL of PBS and lysed by three cycles of freezing/thawing in liquid N_2_/37 °C. Lowry’s protein measurement was then performed. For the determination of CoQ10, 50 µL of CoQ9 (0.2 mg/L, internal standard) and 50 µL of 2 mg/mL p-benzoquinone were added to 100 µL of a fibroblast suspension. After 15 min incubation at room temperature, 850 µL of 1-propanol was added to the fibroblast suspension and centrifuged (12,000 rpm for 15 min at 4 °C). Supernatants were transferred to a glass tube and evaporated to dryness under an N_2_ stream. Dried extracts were then resuspended in a water—1-propanol (2:8) solution. A calibration curve was prepared with 0.2 mg/L CoQ9 internal standard solution and concentrations of CoQ10 ranging from 0.002 to 1 µg/mL. Samples were injected into an Agilent 1290/AB Sciex 4500 LC/MS/MS device. CoQ9 and CoQ10 were separated using a Symmetry C18 HPLC column (Waters, Milford, MA, USA) with a 2-propanol/methanol/formic acid (50:50:0.1) mobile phase and acquired by multiple reaction monitoring in positive mode (CoQ9: 796/197, CoQ10: 864/197).

#### 2.3.3. Cellular Oxygen Consumption

The cellular oxygen consumption rate (OCR) was measured using an XF24 Extracellular Flux Analyzer (Seahorse Bioscience, Izasa Scientific) as previously described [26], except that 60,000 fibroblasts per well were seeded in XF 24-well cell culture microplates and 1 h before the assay the growth medium was replaced with 700 μL of un-buffered fresh MEM medium with 0.5% FBS. After taking an OCR baseline measurement, 50 μL of oligomycin, carbonyl cyanide-4-(trifluoromethoxy) phenylhydrazone (FCCP), rotenone and antimycin A solutions were sequentially added to each well to reach final working concentrations 6 μM, 20 μM, 1 μM and 1 μM respectively. Basal respiration was measured without substrates. Oxygen consumption coupled to ATP production (ATP-linked) was calculated as the difference between basal respiration and the proton leak state determined after the addition of oligomycin. Maximum respiration was measured by stepwise 20 μM titrations of FCCP and inhibition by rotenone and antimycin. Spare capacity was calculated as the difference between maximum and basal respiration.

#### 2.3.4. Mitochondrial Mass and Membrane Potential

Mitochondrial mass and mitochondrial membrane potential were determined by flow cytometry using a BD FACSCanto II flow cytometer (BD Biosciences, San Jose, CA, USA). Cells were loaded with 50 nM MitoTracker green (MitoGreen, 37 °C, 30 min) (Invitrogen, Carlsbad, CA, USA) or 200 nM TMRM (tetramethylrhodamine methyl ester, 37 °C, 30 min) (Thermo Fisher Scientific, Waltham, MS, USA). Data were acquired using FACSDiva software (BD Biosciences, Franklin Lakes, NJ, USA). In each analysis, 10,000 events were recorded.

#### 2.3.5. Mitochondrial Isolation and Western Blotting

Mitochondria were isolated using the hypotonic swelling procedure as previously described [27]. Human dermal fibroblasts were harvested, resuspended in ice-cold isolation buffer (75 mM mannitol, 225 mM sucrose, 10 mM MOPS, 1 mM EGTA and 2 mM PMSF, pH 7.2) and subjected to centrifugation at 1000× *g* for 5 min at 4°C. The cell pellet was then resuspended in cold hypotonic buffer (100 mM sucrose, 10 mM MOPS, 1 mM EGTA and 2 mM PMSF, pH 7.2; 5 mL of buffer/g of cells), homogenized in a Dounce glass homogenizer and incubated on ice for 7 min. Cold hypertonic buffer (1.25 M sucrose and 10 mM MOPS) at 1.1 mL/g of cells and twice the cell-mix volume of isolation buffer plus 2 mg/mL of bovine serum albumin (BSA), were then added to the cell suspension. Cell debris was removed by centrifugation at 1000× *g* for 10 min. Mitochondria were then collected by further centrifugation at 10,000× *g* for 10 min at 4 °C. The pellet was resuspended in isolation buffer without BSA and quantified by Bradford analysis. Mitochondria were denatured in Laemmli buffer for 5 min at 50 °C. The samples were separated by SDS-PAGE and analysed by Western blotting as previously described [28]. The primary polyclonal antibodies used were—anti-total OxPhos (CI-NDUFB8, CII-SDHB, CIII-UQCRC2, CIV-MTCOI and CV-ATP5A) (ab110413; Abcam, Cambridge, UK) at a dilution of 1:250, anti-SDHA (1:5000, ab14715), anti-GFM1 (1:1000, ab173529, Abcam) and anti-MTCO1 (1:1000, ab14705). Anti-GAPDH (ab8245, Abcam) and anti-citrate synthase (C5498, Sigma) at 1:5000 were used as loading controls. Quantitative changes in band intensity were evaluated by densitometry scanning using a calibrated GS-800 densitometer (Bio-Rad, Hercules, CA, USA).

#### 2.3.6. Transmission Electron Microscopy

Electron microscopy imaging of cells was performed as previously described [29] using a Jeol JEM-1010 (JEOL Ltd, Tokyo, Japan) electron microscope operating at 80 kV. Images were recorded with a 4k CMOS F416 camera (TVIPS, Gauting, Germany). For the morphometric analysis of mitochondria, the major and minor axes were measured of at least 50 mitochondria randomly selected from cells as previously described [29]. The aspect ratio was defined as the major axis/minor axis [30]. The minimum aspect ratio of 1 corresponded to a perfect circle.

#### 2.3.7. Minigene Analysis and Morpholino Assay

For the in vitro evaluation of splicing alterations, a fragment of human *GFM1* was cloned into a pSPL3 minigene (Gibco BRL, Carlsbad, CA, USA). For this, gene fragments corresponding to 813 bp of intron 5 of human *GFM1* from patient or control fibroblasts were cloned into the pGEMT easy vector (Promega, Madison, WI, USA). The inserts were then excised with the restriction enzyme EcoRI and cloned into pSPL3. Automated DNA sequencing identified clones containing normal and mutant inserts in the correct orientation. Two micrograms of the wild-type or mutant minigene were transfected into COS7 using the JetPEI reagent (Polyplus Transfection, Illkirch, France). At 24 h post-transfection, the cells were harvested by trypsinization and the RNA purified with trizol. Splicing minigene-derived transcripts were amplified and sequenced using the pSPL3-specific primers SD6 and SA2.

For the morpholino assay, a 25-mer morpholino (5’-GATCACAATGCCATTCGCTCACCTG-3’) targeting NM_024996.5 *GFM1* c.689+908G>A was designed, synthesized and purified by Gene Tools (Oregon, USA). NM_000531.5, a 25-mer morpholino against *OTC* (ornithine carbamoyltransferase), was used as a negative control. The Endo-Porter^®^ delivery reagent (Gene Tools,) was used aid in the transfection following the manufacturer’s recommendations. Some 250,000 fibroblasts from patient Pt16 and from controls were seeded in a P100 and transfected with 0, 10, 20 or 30 µM of morpholino. At 24 h, the cells were harvested for the extraction of total RNA and protein.

#### 2.3.8. Statistical Analysis

Values are expressed as means ± SEM of ‘n’ independently performed experiments in cultured cells. Differences between means were examined using the Student t test. Significance was set at *p* < 0.05. All calculations were performed using GraphPad Prism 6 (GraphPad Software, La Jolla, CA, USA)

## 3. Results

### 3.1. Biochemical Profile

The 39 individuals included in the study represent a heterogeneous patient population with a clinical suspicion of congenital lactic acidosis (neonatal or early childhood onset). Table S1 shows the main clinical features for each patient, annotated using Human Phenotype Ontology (HPO) terms.

For metabolic profiling, amino acid, organic acid and acyl-carnitine metabolomics were examined. Blood lactic acid concentrations at diagnosis ranged from 3.7 to 30 mM and an ≥2X increase in alanine was detected in 10 samples. Urinary organic acids were very consistently raised, with increases in α-hydroxybutyrate detected in 25 urine samples, para-hydroxy-phenyl-derivatives (4-OHphenyl-lactic, 4-OHphenyl-pyruvate or 4-OHphenyl-acetic acids) detected in 19 samples and TCA cycle intermediates detected in 14. Other metabolites such as 3-OH propionic, 3-OH isovaleric or methyl-citrate, 2- or 3-OHglutaric, 3-methylglutaric (3MGA) and 3-methylglutaconic (3-MGC) acids and dicarboxylic acids such as adipic 2-OH and 2-keto adipic acid, also appeared increased although less so. Finally, plasma acyl-carnitines of different chain-lengths showed increases over normal in 10 out of the 26 samples analysed. Since most of these metabolites could reflect immediate or downstream disturbances related to a redox-unbalance compatible with mitochondrial dysfunction, patients were considered to be likely suffering a mitochondrial disorder. To arrive at this result, patients’ biochemical and clinical data were used to score the likelihood of mitochondrial disease being present according to the modified [9] Nijmegen system (mitochondrial disease criteria (MDC)) [2]. Scores of ≥8 indicate a definite disorder, 5–7 a probable disorder, 2–4 a possible disorder and below 2 no disorder (Table S2). The MDC distribution was as follows—36% (14/39) had a definite disorder, 46% returned a score indicating a probable disorder (18/39) and 15% (6/39) a score indicating a possible disorder. One patient remained unclassified (missing data). Neither increases in the metabolites thought linked to disturbances of mitochondrial fatty acid β-oxidation (such as acyl-carnitines), nor 3-OH isovaleric or 3-OH propionic related to branched-chain amino acid catabolism, nor muscle histology, nor OxPhos proteins were contemplated in the above MDC scoring.

### 3.2. Genetic Analysis

The genetic analysis followed three steps—(1) massive parallel sequencing of the clinical exome to identify pathogenic mutations in nuclear genes, (2) mitochondrial DNA analysis, (3) RNA analysis.

#### 3.2.1. DNA Sequencing of Nuclear Genes

In 24 of the 39 patients analysed, massive-parallel sequencing of the clinical exome identified 33 nucleotide sequence variations in 19 different nuclear genes. Seventeen corresponded to genes known to cause CLA—*PDHA1* and *PDHX,* related to pyruvate metabolism; *PHKA2* related to glycogen storage diseases; *ACAD9*, *BCS1L*, *DGUOK*, *COQ2*, *FOXRED1*, *FARS2*, *GFM1*, *MRPS22*, *PDSS1*, *TMEM70*, *TRMU* and *TSFM*, all responsible for primary mitochondrial diseases; and *DLD* and *SLC19A3* related to multiple mitochondrial enzyme complex deficiencies. The remaining two were in the non-CLA-related genes *NPHS2*, responsible for nephrotic syndrome type 2 and *SLC16A1*, which encodes a monocarboxylate transporter (MCT1), that mediates the movement of lactate and pyruvate across cell membranes. In total, seven patients had homozygous variants and 11 more were potentially compound heterozygous. A further three patients carried hemizygous (two boys) or heterozygous (one girl) mutations in the X-linked genes *PHKA2* or *PDHA1* (Table 1). This first massive parallel sequencing analysis also returned three patients (Pt16, Pt19 and Pt21) with a single nucleotide change in three other genes—*GFM1*, *DLD* and *PDHX*—Likely to be involved with CLA. Sanger sequencing validated all the nucleotide changes identified by NGS and confirmed the segregation pattern in family members when samples were available.

Of the 33-nucleotide sequence variations identified, 19 appeared in the Human Gene Mutation Database (HGMD, professional version 2019.2) (https://portal.biobase-international.com/hgmd/pro/all.php); the other 14 were novel. Table 2 lists the variants identified after NGS analysis along with the criteria for their classification according to the joint consensus recommendation of the American College of Medical Genetics and Genomics (ACMG) and the Association for Molecular Pathology [31]. For all novel changes, the in-silico predictions were inconclusive (Table 2 and Table S4).

For the three patients with single heterozygous changes, an extended genomic analysis of large heterozygous deletions was performed using Integrative Genomics Viewer (IGV) software v2.3.98 to analyse the reads of candidate genes visually, along with high-density genotyping. For Pt21, high-density SNP array analysis identified, in heterozygous fashion, a large deletion in chromosome 11 region q14.1 (g.34984192-34988219, Gh37) encompassing part of intron 5–6 and exon 6 of *PDHX*. This was also detected in mRNA analysis (r.642_816del). No large deletions were identified in either Pt16 or Pt19.

#### 3.2.2. Whole Mitochondrial DNA Analysis

Whole mtDNA sequencing was performed for all patients with no putative genetic diagnosis. The deep coverage inherent to this next-generation sequencing system enabled the detection of low-level heteroplasmy. In two of the analysed patients, we identified two previously reported changes—the m.8719G>A (p.Gly65Ter) in *MT-ATP6* that was found in homoplasmy and the m.13513G>A (p.Asp393Asn) in *MT-ND5* gene that results in a 28% of heteroplasmy (Table 1).

In summary, the nuclear (by clinical exome) and mitochondrial DNA analyses returned 24 patients with likely causative changes in either nuclear (22) or mitochondrial genes (2). Two other patients showed a single variation in a strong candidate gene. For the remaining 13 patients, no genetic cause could be identified.

#### 3.2.3. RNA Analysis Helped Diagnose Patients and Can Be Used as Proof of Concept of Personalized Therapies

To complement the exome-based molecular analysis and to improve the interpretation of certain genetic variants, two patients’ RNAs (Pt15 and Pt16) were scanned for possible aberrant transcripts. Pt15 had two single nucleotide variants in *FARS2* gene, the c.737C>T (p.Thr246Met) (predicted as benign) and the previously described c.1082C>T (p.Pro361Leu) but DNA analysis could not confirm the presence on different alleles; Pt16 bore the heterozygous c.2011C>T change in exon 16 of *GFM1;* the second variant was unknown. The lack of fibroblasts for Pt19 hampered any viable *DLD* RNA analysis.

**Figure 1:**
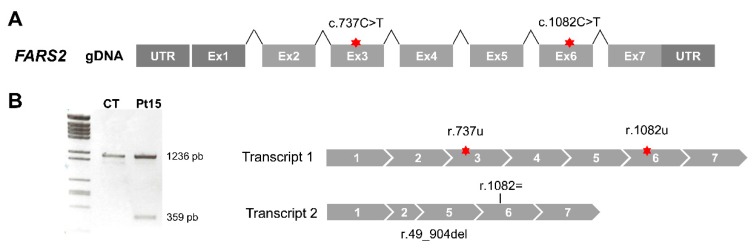
Aberrant splicing of *FARS2* in Pt15. (**A**) Diagram of the human *FARS2* gene. Red stars depict the location of nucleotide variants identified. (**B**) Agarose gel showing the results of reverse transcription polymerase chain reaction (RT-PCR) amplifications in control (CT) and patient (Pt) fibroblasts.

For Pt15 (Figure 1A), cDNA analysis detected a full-length transcript containing both point changes in homozygous fashion (transcript 1) and another smaller transcript (r.49_904del) that skipped the region encompassing part of exon 2 to exon 5 (transcript 2) (Figure 1B). Thus, the genotype at the mRNA level was r.(737c>u;1082c>u); (49_904del).

**Figure 2:**
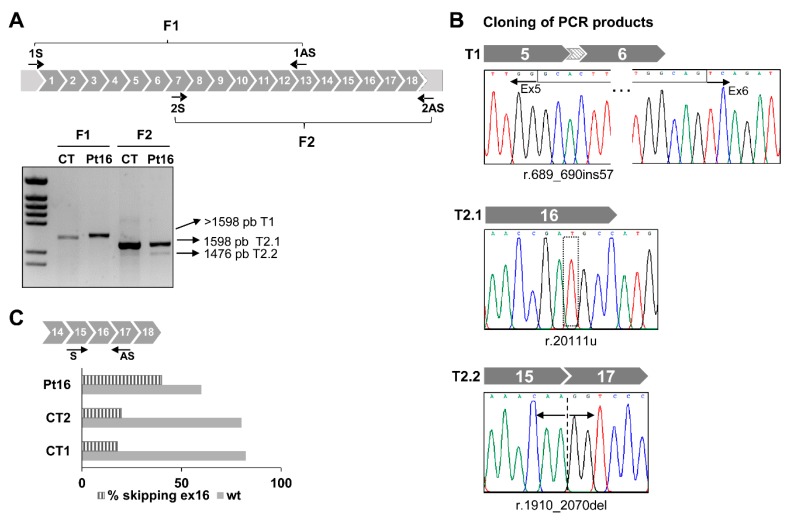
Aberrant splicing of *GFM1* in Pt16. (**A**) Diagram of *GFM1* cDNA with primers (arrows) used to amplify the complete coding region in two overlapping (F1 and F2) fragments. Agarose gel showing the results of RT-PCR amplifications in control (CT) and patient (Pt) fibroblasts. (**B**) Cloning of F1 and F2 PCR products and Sanger sequencing of regions around the nucleotide variations detected. (**C**) Distribution of reads. Data represent the percentage of *GFM1* transcript reads with exon 16 skipped (stripped bars) and full length (filled bars). Read numbers were 41,758 for Pt16, 13,581 for CT1 and 16,718 for CT2.

For Pt16, Sanger sequencing of the RT-PCR products generated using primer combinations to amplify the complete coding sequence in two overlapping fragments (F1 and F2) (Figure 2A) detected a complex profile compatible with the presence of different transcripts. Subsequent sequence analysis of the cloned RT-PCR products confirmed the presence of different transcripts (Figure 2B). Transcript 1 (from the F1 fragment) contained 57 bp of the intronic sequence between exons 5 and 6 of *GFM1*. Transcript 2.1 (from the F2 fragment) was full-length and contained the variant r.2011t (F2.1). Transcript 2.2 (from F2 fragment) skipped exon 16.

Computational tools used to analyse the impact of the change c.2011C>T on splicing events predicted a loss of an enhancer site and the activation of a silencer that would affect the binding of splicing regulatory factors SF2/ASF at position c.2007 resulting in the splicing of exon 16. The massive sequencing of cDNA and the quantization of total reads corresponding to transcripts with junction 15-17 (skipped transcript T2.2) and to full-length transcripts from controls and Pt16 fibroblasts, showed a doubling of the presence of the aberrant T2.2 in the latter patient’s cells. Low levels of the skipped isoform were detected in control fibroblasts (Figure 2C). If the product of this mutant transcript, r.(1910_2070del) were translated it would lead to a frameshift creating a premature stop codon, p.(Ala637Glyfs*5), with a likely impact on EFG1 protein synthesis or stability.

The origin of the aberrant transcript containing 57 bp of the intronic sequence of intron 5 of *GFM1* was also determined and the change c.689+908G>A identified at DNA level. This change was previously proposed as responsible for the activation of a cryptic splice site and the creation of a new exon already present in the *GFM1* isoform ENST00000264263.9 (Figure S1A) [32]. Segregation pattern analysis of the progenitors’ DNA corroborated the presence of c.689+908G>A and c.2011C>T in different alleles (Figure S1B). The final confirmation of the direct involvement of change c.689+908G>A in the inclusion of the 57 bp intronic sequence was obtained by minigene analysis using a recombinant pSPL3 construct containing 813 bp of the intronic 5-6 sequence. Transcriptional profile analysis of the recombinant mutant plasmid showed the 57 bp transcript seen in patient Pt16 fibroblasts (Figure S1C). No pseudo-exon inclusion was detected in control cells. Western blot analysis of the EGF1 protein encoded by *GFM1* in patient Pt16’s cells showed a drastically reduced amount of protein (Figure S1D).

#### 3.2.4. Antisense Oligonucleotide Treatment Rescues the Aberrant Splicing Event Caused by the Intronic Variant GFM1 c.689+908G>A

**Figure 3:**
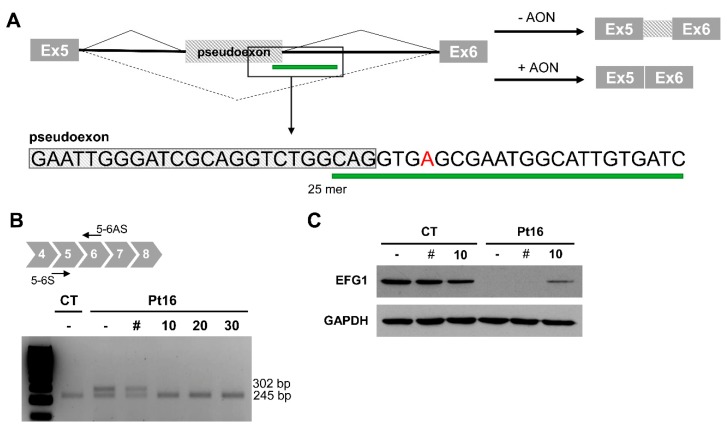
Antisense morpholino oligonucleotide-based pseudoexon skipping efficacy. (**A**) Diagram of the pseudoexon insertion caused by c.689+908G>A and the predicted effect of the antisense morpholino oligonucleotide (AON). Inset showing location and sequence of the 25mer AON. (**B**) Representative image of the RT-PCR product from mutant (Pt16) and wild type (CT) cells, non-transfected (-) and transfected with 10 μM of non-target control (#); or in the presence of different concentrations (0 to 30 μM) of *GFM1*-specific AON. (**C**) EFG1 rescue upon treatment with 10 μM of non-target control (#) or *GFM1*-specific AON.

In an attempt to block mutant pre-mRNA access to the splicing machinery and to try to circumvent the formation of the aberrantly spliced transcript associated with change c.689+908G>A, tests were made of the ability of an antisense morpholino oligonucleotide (AON) to overlap the c.689+908G>A variation (Figure 3A). Transfection with 10 μM of the AON restored the correct splicing of *GFM1* in Pt16 fibroblasts carrying the deep-intronic variant in heterozygous fashion (Figure 3B). The AON did not alter the normal splicing of *GFM1*. Upon AON delivery, the level of EGF1 was partially restored as should correspond to the heterozygous condition of the intronic change (Figure 3C).

### 3.3. Functional Studies in Patients with Novel Genotypes

#### 3.3.1. Biochemical Confirmation of Genetic Data

The pathogenicity of the changes in *DLD* carried by patients Pt18 and Pt35 was assessed by the direct measurement of dihydrolipoamide dehydrogenase activity in fibroblasts (see Table S2). The activity in the patients’ cells was respectively 3% and 10% that of control cells.

CoQ10 levels were measured in the fibroblasts of patients Pt9 and Pt10, who carried changes in *COQ2* and *PDSS1* (both of which code for enzymes in the CoQ10 synthesis pathway). Reductions of 76% (11.9 ± 1.3 pmol/mg prot) were recorded for Pt9 and of 91.5% (4.2 ± 1.5 pmol/mg prot) for Pt10, both compatible with the available genotype data. Control values were 49.6 ± 10.8 pmol/mg protein.

#### 3.3.2. Mitochondrial Respiration and OxPhos Protein Analysis Confirmed Mitochondrial Dysfunction

**Figure 4:**
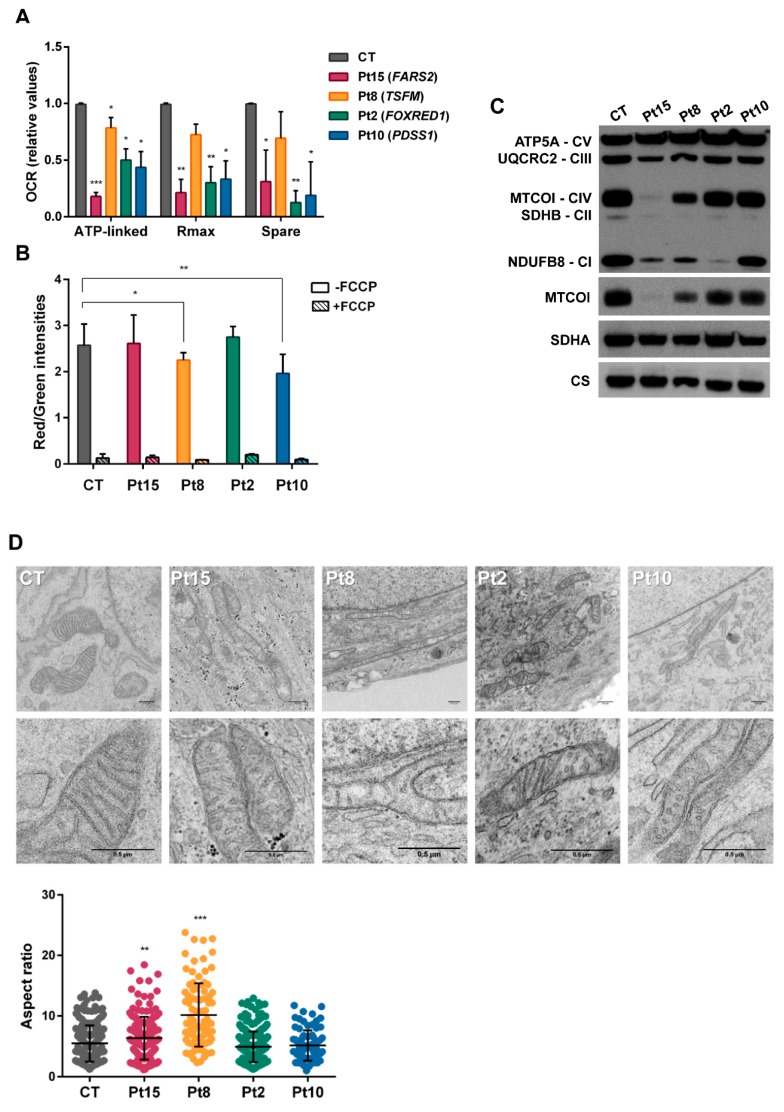
Bioenergetics of congenital lactic acidosis (CLA)-patients’ fibroblasts. (**A**) Oxygen consumption rates. The data shown are for ATP-production-dependent maximal respiration (Rmax) and spare capacity (spare). Results are expressed as fold over the control concentrations and are the mean ± SD of 3–5 wells from *n* = 2–3 independent experiments. Control values are the means of two different control cell lines. (**B**) Flow cytometry analysis of mitochondrial mass (Mitotracker green) and membrane potential (TMRM staining) in the absence/presence of carbonyl cyanide-4-(trifluoromethoxy) phenylhydrazone (FCCP). Results are the means of three independent experiments. (**C**) Western blots for representatives of all five respiratory complexes. Anti-MTOC1, anti-SDHA and anti-citrate synthase were also included. (**D**) Electron microscopy images showing defects of mitochondrial ultrastructure and cristae organization in patient fibroblasts. Mitochondrial length was analysed in control (CT) and patient (Pt) fibroblasts. Mitochondrial enlargement is expressed as the aspect ratio (major/minor mitochondrial axis ratio). Student *t* test (* *p* < 0.05; ** *p* < 0.01; *** *p* < 0.001).

For patients Pt15, Pt8, Pt2 and Pt10 carrying novel alleles in *FARS2*, *TSFM*, *FOXRED1* and *PDSS1* that could potentially provoke alterations in oxidative phosphorylation, assessments of possible mitochondrial dysfunction were made in terms of alterations in the OCR, mitochondrial membrane potential, OxPhos proteins and mitochondrial ultrastructure. Figure 4A shows a significant reduction detected in the ATP-linked oxygen consumption ratio, maximal OCR and reserve capacity for all patient fibroblasts. The ratio of red (TMRM) to green (MitoTracker^®^green) staining in fibroblasts corroborated a significant reduction in mitochondrial membrane potential in the cells of Pt8 and Pt10 (Figure 4B). In agreement with the oxygen consumption data, SDS-PAGE showed reduction in several OxPhos proteins (Figure 4C). The mitochondrial-encoded MTCO1 protein appeared reduced in mitochondrial extracts from the fibroblasts of patients Pt15 (*FARS2*) and Pt8 (*TSFM*) and the nuclear-encoded subunit of complex I, NDUFB8, was reduced in Pt15 (*FARS2*) and Pt2 (*FOXRED1*). Finally, and contrary to that previously reported [32,33], the mitochondrial extracts of Pt8 (*TSFM*) showed reduced amounts of all representative OxPhos proteins, except for complex V.

Transmission electron microscopy of the fibroblasts of patients Pt15, Pt8, Pt2 and Pt10 (Figure 4D) revealed the predominant presence of loose cristae, a condition compatible with changes in respiration capacity. Patients Pt8 and Pt15 also had a significant number of elongated mitochondria.

## 4. Discussion

This study reports how the combination of targeted-exome DNA sequencing, mtDNA analysis and functional genetics analysis identified genetic variants likely causal of CLA in 25 out of 39 patients (64%). All had a biochemical hallmark of persistent lactic acidosis and metabolites mostly related to downstream effects of an altered NADH/NAD+ redox status [6], the concentrations of which correlated with the blood lactate concentration. Based on their clinical and biochemical data, all patients received MDC scores and their nuclear and mitochondrial DNA was examined to identify genes and nucleotide variations likely responsible for pathological phenotypes. In this way, potentially biallelic changes were identified in 19 nuclear genes, 17 in genes related to congenital lactic acidosis and two in unexpected genes. Another two patients carried changes in their mitochondrial DNA compatible with their disease phenotype [34,35].

Before reporting the results of the diagnostic genetic testing to the corresponding clinicians, several lines of diagnostic evidence were taken into account. One was whether there was a confirmed causal link between a defective gene and patient phenotype. For patients Pt33, Pt5, Pt17, Pt6, Pt4, Pt3 and Pt34 carrying changes in *BCS1L*, *DGUOK*, *GFM1*, *MRPS22*, *TMEM70*, *MT-ATP6*, *MT-ND5*, the changes identified had been previously described associated with mitochondrial disorders and most of them shared phenotypic features with other patients previously reported [36,37,38,39]. The same occurred with patients Pt36, Pt39 who carried changes in *SLC19A3* and patients Pt12, Pt20 and Pt21 who had mutations in genes coding for subunits of the pyruvate dehydrogenase complex. Protein-specific functional analysis provided another line of evidence, as did the direct assay of enzymatic function in dermal fibroblasts. The latter corroborated the disease-causing nature of the nucleotide variations identified in patients Pt18 and Pt35 who carried biallelic changes in *DLD*. Further evidence was supplied by the re-phenotyping of patients to identify specific characteristics associated with defined biochemical phenotypes or by establishing a mitochondrial bioenergetics dysfunction related to the genes and changes identified. Thus, the reduced COQ10 measured in the fibroblasts of patients Pt9 and Pt10 carrying biallelic changes in *COQ2* and *PDSS1* respectively, helped confirm the deficient synthesis of coenzyme COQ10 in their fibroblasts. Because CoQ10 synthesis or thiamine transport defects are treatable conditions in which early diagnosis is essential to improve the clinical outcome [7,40,41], patients with them were immediately treated.

Access to a disease-relevant tissue is a problem in mitochondrial dysfunction analyses but epidermal fibroblasts have been reported to show potential in functional evaluations of many mitochondrial diseases [14,33,42]. In line with its role in electron transport, functional data from the fibroblasts of Pt15, Pt8, Pt2 and Pt10 confirmed the presence of impaired mitochondrial respiration and reduced amounts of representative OxPhos proteins. Thus, the diminished MTCO1 seen in patients carrying nucleotide changes in *FARS2* and *TSFM* reflect the reduced function of the transcription/translation mitochondrial machinery. The reduction in NDUFB8 protein, observed in mitochondrial extracts from patients with changes in *FOXRED1*, *FARS2* or *TSFM* might reflect the instability of this protein when not incorporated into OxPhos complex I [43]. Similar patterns have been reported in other patients with mutations in *FARS2* and *FOXRED1* [42,44]. Finally, the ultrastructure and morphology of the mitochondria and their cristae agreed with a possible alteration in mitochondrial bioenergetics function.

The present exome analysis also returned some unexpected results. For example, in patients Pt11 and Pt22, who met the criteria for mitochondrial disease with relatively high MDC scores, DNA analysis identified biallelic changes in *NPHS2* and *SLC16A1*, which might be a phenocopy of mitochondrial disease. In Pt22, respirometer tests (Figure S2) detected a notable decline in fibroblast respiratory variables compatible with mitochondrial dysfunction. However, not even whole exome sequencing returned positive results in terms of putative mutations related to mitochondrial disorders (data not shown).

The present work detected one patient, Pt16, with a single variation in a strong candidate gene and another patient, Pt15 with a possible deficiency in *FARS2* but with a nucleotide variant predicted to be benign and that did not correlate with the severity of the existing protein defect. In both patients, transcript analysis identified aberrant splicing events, probably due to disruptions of the consensus splice-site signals or to an effect on the splicing elements within the pre-mRNA. This analysis also returned important results concerning the relationship between genotype and phenotype. Thus, for patient Pt15, the new genotype deciphered by transcriptomic analysis better matched the drastic mitochondrial dysfunction observed in this patient’s fibroblasts. For Pt16, in addition to characterizing the second mutation in the *GFM1* gene, an aberrant and probably unstable mRNA that skipped exon 16 was identified associated to the c.2011C>T (p.Arg671Cys) change. If translated it would produce a truncated p.(Ala637Glyfs*5) protein. Until this finding was made, the most plausible cause of the total absence of EFG1 in the fibroblasts of patients carrying the p.Arg671Cys change [33,39] was the disruption of the inter-subunit interface of the protein; this would locally destabilize the mutant protein resulting in its absence [45]. The present results, which increase the number of variants within exons causing disruptions in normal mRNA processes [46], shed new light on the effect of the nucleotide change on EFG1 expression and stability and highlight the importance of evaluating the effect of genomic variants on splicing as an integral part of the diagnostic work-up.

Overall, a genetic diagnosis was reached for 25 of the present 39 patients, obviating the need for muscle or hepatic biopsies and so forth. The highest percentage of positive results were obtained in patients with a definite mitochondrial disease, highlighting the importance of combining metabolomic, genetic and phenomic data in arriving at a diagnosis (Table 1). Although in this study, patients were initially selected based on their clinical and biochemical data, the present results support a “genome first approach” [15] be followed. In other words, genetic analyses should come first and the results of biochemical analyses and so forth, should be used in the interpretation of the genetic results.

The present work also reports the efficacy of antisense oligonucleotide therapy for rescuing normal splicing of the cryptic splice site generated by *GFM1* c.689+908G>A in patient fibroblasts. Easy to design and highly specific, antisense oligonucleotides have been used as RNA-modulators in cellular models of several genetic disorders [47] including ISCU myopathy [48]. The challenge is always to achieve the safe and efficient delivery of the therapeutic oligonucleotide to the required tissues.

## 5. Conclusions

The discussed system facilitates the diagnosis of CLA while greatly restricting the need to use invasive techniques and increases our knowledge of the causes of this condition. Identification of the genetic cause can also facilitate genetic counselling and guide the design of personalized therapeutic strategies.

## Figures and Tables

**Table 1:** Genes and variants.

Patient	MDC	Inheritance	Gene	Phenotype MIM Number	RefSeq/Variant 1	RefSeq/Variant 2
Pt1.1 * **Pt1.2**	Definite	AR	*ACAD9*	# 611126 Mitochondrial Complex I Deficiency Due To ACAD9 Deficiency	NM_014049.4: c.359delT (p.Phe120Serfs*9)	NM_014049.4: c.473C>T (p.Thr158Ile)
Pt2 *	Definite	AR	*FOXRED1*	# 256000 Leigh Syndrome Due To Mitochondrial Complex I Deficiency	NM_017547.3: c.628T>G (p.Tyr210Asp)	NM_017547.3: c.1273C>T (p.His425Tyr)
**Pt3**	Definite	Mit	*MT-ATP6*	# 516060 Mitochondrial Complex V(ATP Synthase) Deficiency	NC_012920.1: m.G8719A (p.Gly65 *)	
Pt4 *	Definite	AR	*TMEM70*	# 614052 Mitochondrial Complex V (ATPSynthase) Deficiency, Nuclear Type 2	NM_017866.5: c.317-2A>G	NM_017866.5: c.317-2A>G
**Pt5**	Definite	AR	*DGUOK*	# 251880 Mitochondrial DNA Depletion Syndrome 3 (Hepato-cerebral Type)	NM_080916.2: c.763_766dupGATT (p.Phe256*)	NM_080916.2: c.763_766dupGATT (p.Phe256*)
**Pt6**	Definite	AR	*MRPS22*	# 611719 Combined Oxidative Phosphorylation Deficiency 5	NM_020191.2: c.509G>A (p.Arg170His)	NM_020191.2: c.1032_1035dupAACA (p.Leu346Asnfs*21)
Pt7 *	Definite	AR	*TRMU*	# 613070 Liver Failure, Infantile, Transient	NM_018006.4: c.680G>C (p.Arg227Thr)	NM_018006.4: c.1041_1044dupTCAA (p.Asp349Serfs*58)
**Pt8**	Definite	AR	*TSFM*	# 610505 Combined Oxidative Phosphorylation Deficiency 3	NM_001172696.1: c.782G>C (p.Cys261Ser)	NM_001172696.1: c.848G>A (p.Gly283Asp)
Pt9 *	Definite	AR	*COQ2*	# 607426 Coenzyme Q10 Deficiency, Primary, 1	NM_015697.7: c.163C>T (p.Arg55 *)	NM_015697.7: c.1197delT (p.Asn401Ilefs*15)
Pt10 *	Definite	AR	*PDSS1*	# 614651 Coenzyme Q10 Deficiency, Primary, 2	NM_014317.3: c.716T>G (p.Val239Gly)	NM_014317.3: c.1183C>T (p.Arg395*)
Pt11 *	Definite	AR	*NPHS2*	# 600995 Nephrotic Syndrome, Type 2	NM_014625.3: c.413G>A (p.Arg138Gln)	NM_014625.3: c.413G>A (p.Arg138Gln)
**Pt12**	Definite	XL	*PDHA1*	# 312170 Pyruvate Dehydrogenase E1-Alpha Deficiency	NM_000284.3: c.787C>G (p.Arg263Gly)	
**Pt15**	Probable	AR	*FARS2*	# 614946 Combined Oxidative Phosphorylation Deficiency 14	NM_006567.3: c.737C>T (p.Thr246Met)	NM_006567.3: c.1082C>T (p.Pro361Leu)
Pt16 *	Probable	AR	*GFM1*	# 609060 Combined Oxidative Phosphorylation Deficiency 1	NM_024996.5: c.2011C>T (p.Arg671Cys)	a.NM_024996.5: c.689+908G>A r.(689_690ins57) (p.Gly230_231Glnins19)
Pt17 *	Probable	AR	*GFM1*	# 609060 Combined Oxidative Phosphorylation Deficiency 1	NM_024996.5: c.2011C>T (p.Arg671Cys)	NM_024996.5: c.1404delA (p.Gly469Valfs*84)
**Pt18**	Probable	AR	*DLD*	# 246900 Dihydrolipoamide Dehydrogenase Deficiency	NM_000108.4: c.259C>T (p.Pro87Ser)	NM_000108.4: c.946C>T (p.Arg316*)
**Pt19**	Probable	AR	*DLD*	# 246900 Dihydrolipoamide Dehydrogenase Deficiency	NM_000108.4: c.788G>A (p.Arg263His)	not found
Pt20 **	Probable	XL	*PDHA1*	# 312170 Pyruvate Dehydrogenase E1-Alpha Deficiency	NM_000284.3: c.506C>T (p.Ala169Val)	=
**Pt21**	Probable	AR	*PDHX*	# 245349 Pyruvate Dehydrogenase E3-Binding Protein Deficiency	NM_003477.2: c.965-1G>A (p.Asp322Alafs*6)	b.NG_013368.1: g.34984192_34988219del r.642_816del (p.Asp215Alafs*19)
Pt22 *	Probable	AR	*SLC16A1*	# 616095 Monocarboxylate Transporter 1 Deficiency	NM_003051.3: c.747_750delTAAT (p.Asn250Serfs*5)	NM_003051.3: c.747_750delTAAT (p.Asn250Serfs*5)
**Pt23**	Probable	XLR	*PHKA2*	# 306000 Glycogen Storage Disease IXA1	NM_000292.2: c.1246G>A (p.Gly416Arg)	
**Pt33**	Possible	AR	*BCS1L*	# 124000 Mitochondrial Complex III Deficiency, Nuclear Type 1	NM_004328.4 c.166C>T (p.Arg56*)	NM_004328.4 c.-147A>G (p.?)
**Pt34**	Possible	Mit	*MT-ND5*	# 540000 Mitochondrial Myopathy, Encephalopathy, Lactic Acidosis and Stroke-Like Episodes	NC_012920.1: m.13513G>A (p.Asp393Asn)	
Pt35 *	Possible	AR	*DLD*	# 246900 Dihydrolipoamide Dehydrogenase Deficiency	NM_000108.4 c.647T>C (p.Met216Thr)	NM_000108.4 c.647T>C (p.Met216Thr)
Pt36 *	Possible	AR	*SLC19A3*	# 607483 Thiamine Metabolism Dysfunction Syndrome 2 (Biotin- Or Thiamine-Responsive Type)	NM_025243.3: c.20C>A (p.Ser7*)	NM_025243.3: c.20C>A (p.Ser7*)
Pt39 *		AR	*SLC19A3*	# 607483 Thiamine Metabolism Dysfunction Syndrome 2 (Biotin- Or Thiamine-Responsive Type)	NM_025243.3: c.20C>A (p.Ser7*)	NM_025243.3: c.20C>A (p.Ser7*)

Abbreviations: * Mendelian segregation confirmed; ** Not found in mother; Mitochondrial disease criteria (MDC); Autosomal recessive (AR); X-Linked (XL); X-Linked recessive (XLR); Mitochondrial DNA (Mit). In bold, nucleotide variations identified after complementary test; ^a^ RNA analysis; ^b^ SNP array and RNA analysis. The mutation nomenclature follows that used by Mutalyzer 2.0.29 (https://mutalyzer.nl/). RefSeq number for each gene is included.

**Table 2:** Analysis of variants identified by massive-parallel sequencing and pathogenicity status.

Gene	Variant/ Consequence	ACMG Tags	Classification	HGMD Accession	gnomaD
*ACAD9*	c.359delT (p.Phe120Serfs*9)	PVS1, PM2, PP1, PP5	Pathogenic	CD153914	0.0001042
*ACAD9*	c.473C>T (p.Thr158Ile)	PM2, PM3, PP1, PP3	Likely pathogenic	-	0
*BCS1L*	c.166C>T (p.Arg56Ter)	PS3, PM4, PP3, PP5	Likely pathogenic	CM022763	0.0001626
*BCS1L*	c.-147A>G (p.?)	PM2 PM3, PP5	VUS	CS098028	0
*COQ2*	c.1197delT (p.Asn401Ilefs*15)	PM2, PM3, PM4, PP5	Likely pathogenic	CD071308	1.217e-5
*COQ2*	c.163C>T (p.Arg55Ter)	PM2, PM3, PM4, PP1 PS3	Likely pathogenic	-	0
*DGUOK*	c.763_766dupGATT (p.Phe256Ter)	PM4, PM2, PM5, PS3, PP5	Likely pathogenic	CI034484	2.031e-5
*DLD*	c. 259C>T (p.Pro87Ser)	PM2, PM3, PP2, PP3, PP4	VUS	-	0
*DLD*	c.647T>C (p.Met216Thr)	PM2, PP2, PP3	VUS	-	0
*DLD*	C.788G>A (p.Arg263Hys)	PM3, PP3	VUS	-	0.000817
*DLD*	c.946C>T (p.Arg316Ter)	PM2, PM4, PP3, PP4	Likely pathogenic	-	1.63e-05
*FARS2*	c.737C>T (p.Thr246Met)	BS2, BP6	Likely benign	-	0.004064
*FARS2*	c.1082C>T (p.Pro361Leu)	PP3	VUS	CM1718796	0.0001339
*FOXRED1*	c.628T>G (p.Tyr210Asp)	PM2, PP3	VUS	-	0
*FOXRED1*	c.1273C>T (p.His425Tyr)	PM2, PP3	VUS	-	0
*GFM1*	c.1404delA (p.Gly469Valfs*84)	PVS1, PM2, PP5	Pathogenic	CD154422	1.635e-5
*GFM1*	c.2011C>T (p.Arg671Cys)	PM2, PM3, PP2, PP3, PP5	Likely pathogenic	CM11881	7.216e-5
*MRPS22*	c.1032_1035dupAACA (p.Leu346Asnfs*21)	PVS1, PM3, PP5	Pathogenic	CI152171	9.028e-5
*MRPS22*	c.509G>A (p.Arg170His)	PM2, PM3, PP3, PP5	Likely pathogenic	CM076316	7.221e-5
*NPHS2*	c.413G>A (p.Arg138Gln)	PP2, PP3, PP5	VUS	CM000581	0.0005739
*PDHA1*	c.506C>T (p.Ala169Val)	PM2, PP2, PP3, PP5	VUS	CM091028	0
*PDHA1*	c.787C>G (p.Arg263Gly)	PM2, PM5, PS3, PS4, PP2, PP3, PP5	Pathogenic	CM920573	0
*PDHX*	c.965-1G>A (p.Asp322Alafs*6)	PVS1, PM2, PM3, PP3, PP5	Pathogenic	CS024024	4.11e-6
*PDSS1*	c.716T>G (p.Val239Gly)	PM2, PP3 (PS3 - Likely path)	VUS	-	0
*PDSS1*	c.1183C>T (p.Arg395Ter)	PM2, PM4, PP3	Pathogenic	-	4.064e-6
*PHKA2*	c.1246G>A (p. Gly416Arg)	PP3, BP6	VUS	-	0.00432
*SLC16A1*	c.747_750delTAAT (p.Asn250Serfs*5)	PVS1, PM2, PP5	Pathogenic	CD1411339	8.123e-6
*SLC19A3*	c.20C>A (p.Ser7Ter)	PM2, PM3, PM4, PP4, PP5	Likely pathogenic	CM131528	0
*TMEM70*	c.317-2A>G (p.?)	PVS1, PM2, PP1, PP5	Pathogenic	CS084884	7.605e-5
*TRMU*	c.1041_1044dupTCAA (p.Asp349Serfs*58)	PVS1, PM2, PP5	Pathogenic	CD155923	1.219e-5
*TRMU*	c.680G>C (p.Arg227Thr)	PM2, PM3, PP3	VUS	-	1.624e-5
*TSFM*	c.782G>C (p. Cys261Ser)	PS3, PM2	Likely pathogenic	CM170018	4.188e-6
*TSFM*	c.848G>A (p. Gly283Asp)	PM2, PP3	VUS	-	5.889e-5

The DNA variant numbering system was based on the cDNA sequence. Nucleotide numbering uses +1 as the A of the ATG translation initiation codon in the reference sequence, with the initiation codon as codon 1. Tags for classifying missense changes are those according the American College of Medical Genetics and Genomics (ACMG). Classification was accomplished using the VarSome web platform. Accession number from HGMD^®^ Professional 2019.2 (https://portal.biobase-international.com/hgmd/pro/start.php?) and allele frequency from https://gnomad.broadinstitute.org/ are also included.

## Supplementary Materials

The following are available online at https://www.mdpi.com/2077-0383/8/11/1811/s1, Figure S1: Genomic characterization of variant c.689+908G>A and effect of the change on minigene-splicing profile, Figure S2: Oxygen consumption rates of fibroblasts from Pt22, Table S1: Clinical phenotypes. Table S2: Biochemical data, Table S3: Primers used for massive parallel sequencing of *GFM1* cDNA amplicons, Table S4: Prediction of pathogenicity by different tools.

## References

[1] Kraut J.A., Madias N.E. (2014). Lactic acidosis. N. Engl. J. Med..

[2] Morava E., van den Heuvel L., Hol F., de Vries M.C., Hogeveen M., Rodenburg R.J., Smeitink J.A. (2006). Mitochondrial disease criteria: Diagnostic applications in children. Neurology.

[3] Legati A., Reyes A., Nasca A., Invernizzi F., Lamantea E., Tiranti V., Garavaglia B., Lamperti C., Ardissone A., Moroni I. (2016). New genes and pathomechanisms in mitochondrial disorders unraveled by NGS technologies. Biochim. Biophys. Acta.

[4] Gorman G.S., Chinnery P.F., DiMauro S., Hirano M., Koga Y., McFarland R., Suomalainen A., Thorburn D.R., Zeviani M., Turnbull D.M. (2016). Mitochondrial diseases. Nat. Rev. Dis. Prim..

[5] Parikh S., Goldstein A., Karaa A., Koenig M.K., Anselm I., Brunel-Guitton C., Christodoulou J., Cohen B.H., Dimmock D., Enns G.M. (2017). Patient care standards for primary mitochondrial disease: A consensus statement from the Mitochondrial Medicine Society. Genet. Med..

[6] Thompson Legault J., Strittmatter L., Tardif J., Sharma R., Tremblay-Vaillancourt V., Aubut C., Boucher G., Clish C.B., Cyr D., Daneault C. (2015). A Metabolic Signature of Mitochondrial Dysfunction Revealed through a Monogenic Form of Leigh Syndrome. Cell Rep..

[7] Ortigoza-Escobar J.D., Molero-Luis M., Arias A., Oyarzabal A., Darin N., Serrano M., Garcia-Cazorla A., Tondo M., Hernandez M., Garcia-Villoria J. (2016). Free-thiamine is a potential biomarker of thiamine transporter-2 deficiency: A treatable cause of Leigh syndrome. Brain.

[8] Desbats M.A., Lunardi G., Doimo M., Trevisson E., Salviati L. (2015). Genetic bases and clinical manifestations of coenzyme Q10 (CoQ 10) deficiency. J. Inherit. Metab. Dis..

[9] Witters P., Saada A., Honzik T., Tesarova M., Kleinle S., Horvath R., Goldstein A., Morava E. (2018). Revisiting mitochondrial diagnostic criteria in the new era of genomics. Genet. Med..

[10] Rahman J., Noronha A., Thiele I., Rahman S. (2017). Leigh map: A novel computational diagnostic resource for mitochondrial disease. Ann. Neurol..

[11] Wortmann S.B., Koolen D.A., Smeitink J.A., van den Heuvel L., Rodenburg R.J. (2015). Whole exome sequencing of suspected mitochondrial patients in clinical practice. J. Inherit. Metab. Dis..

[12] Kohda M., Tokuzawa Y., Kishita Y., Nyuzuki H., Moriyama Y., Mizuno Y., Hirata T., Yatsuka Y., Yamashita-Sugahara Y., Nakachi Y. (2016). A Comprehensive Genomic Analysis Reveals the Genetic Landscape of Mitochondrial Respiratory Chain Complex Deficiencies. PLoS Genet..

[13] Puusepp S., Reinson K., Pajusalu S., Murumets U., Oiglane-Shlik E., Rein R., Talvik I., Rodenburg R.J., Ounap K. (2018). Effectiveness of whole exome sequencing in unsolved patients with a clinical suspicion of a mitochondrial disorder in Estonia. Mol. Genet. Metab. Rep..

[14] Kremer L.S., Bader D.M., Mertes C., Kopajtich R., Pichler G., Iuso A., Haack T.B., Graf E., Schwarzmayr T., Terrile C. (2017). Genetic diagnosis of Mendelian disorders via RNA sequencing. Nat. Commun..

[15] Raymond F.L., Horvath R., Chinnery P.F. (2018). First-line genomic diagnosis of mitochondrial disorders. Nat. Rev. Genet..

[16] Chalmers R.A., Lawson A.M., Chalmers R.A., Lawson A.M. (1982). Gas Chomatography-Mass spectometry. Organic Acids in Man. Analytical Chemistry, Biochemistry and Diagnosis of the Organic Acidurias.

[17] Ferrer I., Ruiz-Sala P., Vicente Y., Merinero B., Perez-Cerda C., Ugarte M. (2007). Separation and identification of plasma short-chain acylcarnitine isomers by HPLC/MS/MS for the differential diagnosis of fatty acid oxidation defects and organic acidemias. J. Chromatogr. B Analyt. Technol. Biomed. Life Sci..

[18] Vega A.I., Medrano C., Navarrete R., Desviat L.R., Merinero B., Rodriguez-Pombo P., Vitoria I., Ugarte M., Perez-Cerda C., Perez B. (2016). Molecular diagnosis of glycogen storage disease and disorders with overlapping clinical symptoms by massive parallel sequencing. Genet. Med..

[19] Ruiz-Pesini E., Lott M.T., Procaccio V., Poole J.C., Brandon M.C., Mishmar D., Yi C., Kreuziger J., Baldi P., Wallace D.C. (2007). An enhanced MITOMAP with a global mtDNA mutational phylogeny. Nucleic Acids Res..

[20] Weissensteiner H., Forer L., Fuchsberger C., Schopf B., Kloss-Brandstatter A., Specht G., Kronenberg F., Schonherr S. (2016). mtDNA-Server: Next-generation sequencing data analysis of human mitochondrial DNA in the cloud. Nucleic Acids Res..

[21] Kopanos C., Tsiolkas V., Kouris A., Chapple C.E., Albarca Aguilera M., Meyer R., Massouras A. (2018). VarSome: The human genomic variant search engine. Bioinformatics.

[22] Li B., Krishnan V.G., Mort M.E., Xin F., Kamati K.K., Cooper D.N., Mooney S.D., Radivojac P. (2009). Automated inference of molecular mechanisms of disease from amino acid substitutions. Bioinformatics.

[23] Tang H., Thomas P.D. (2016). PANTHER-PSEP: Predicting disease-causing genetic variants using position-specific evolutionary preservation. Bioinformatics.

[24] Bravo-Alonso I., Navarrete R., Arribas-Carreira L., Perona A., Abia D., Couce M.L., Garcia-Cazorla A., Morais A., Domingo R., Ramos M.A. (2017). Nonketotic hyperglycinemia: Functional assessment of missense variants in GLDC to understand phenotypes of the disease. Hum. Mutat..

[25] Oyarzabal A., Martinez-Pardo M., Merinero B., Navarrete R., Desviat L.R., Ugarte M., Rodriguez-Pombo P. (2013). A novel regulatory defect in the branched-chain alpha-keto acid dehydrogenase complex due to a mutation in the PPM1K gene causes a mild variant phenotype of maple syrup urine disease. Hum. Mutat..

[26] Bravo-Alonso I., Oyarzabal A., Sanchez-Arago M., Rejas M.T., Merinero B., Garcia-Cazorla A., Artuch R., Ugarte M., Rodriguez-Pombo P. (2016). Dataset reporting BCKDK interference in a BCAA-catabolism restricted environment. Data Br..

[27] Mohanraj K., Wasilewski M., Beninca C., Cysewski D., Poznanski J., Sakowska P., Bugajska Z., Deckers M., Dennerlein S., Fernandez-Vizarra E. (2019). Inhibition of proteasome rescues a pathogenic variant of respiratory chain assembly factor COA7. EMBO Mol. Med..

[28] Garcia-Cazorla A., Oyarzabal A., Fort J., Robles C., Castejon E., Ruiz-Sala P., Bodoy S., Merinero B., Lopez-Sala A., Dopazo J. (2014). Two novel mutations in the BCKDK (branched-chain keto-acid dehydrogenase kinase) gene are responsible for a neurobehavioral deficit in two pediatric unrelated patients. Hum. Mutat..

[29] Oyarzabal A., Bravo-Alonso I., Sanchez-Arago M., Rejas M.T., Merinero B., Garcia-Cazorla A., Artuch R., Ugarte M., Rodriguez-Pombo P. (2016). Mitochondrial response to the BCKDK-deficiency: Some clues to understand the positive dietary response in this form of autism. Biochim. Biophys. Acta.

[30] De Vos K.J., Allan V.J., Grierson A.J., Sheetz M.P. (2005). Mitochondrial function and actin regulate dynamin-related protein 1-dependent mitochondrial fission. Curr. Biol..

[31] Richards S., Aziz N., Bale S., Bick D., Das S., Gastier-Foster J., Grody W.W., Hegde M., Lyon E., Spector E. (2015). Standards and guidelines for the interpretation of sequence variants: A joint consensus recommendation of the American College of Medical Genetics and Genomics and the Association for Molecular Pathology. Genet. Med..

[32] Simon M.T., Ng B.G., Friederich M.W., Wang R.Y., Boyer M., Kircher M., Collard R., Buckingham K.J., Chang R., Shendure J. (2017). Activation of a cryptic splice site in the mitochondrial elongation factor GFM1 causes combined OXPHOS deficiency. Mitochondrion.

[33] Emperador S., Bayona-Bafaluy M.P., Fernandez-Marmiesse A., Pineda M., Felgueroso B., Lopez-Gallardo E., Artuch R., Roca I., Ruiz-Pesini E., Couce M.L. (2017). Molecular-genetic characterization and rescue of a TSFM mutation causing childhood-onset ataxia and nonobstructive cardiomyopathy. Eur. J. Hum. Genet..

[34] Tang S., Wang J., Zhang V.W., Li F.Y., Landsverk M., Cui H., Truong C.K., Wang G., Chen L.C., Graham B. (2013). Transition to next generation analysis of the whole mitochondrial genome: A summary of molecular defects. Hum. Mutat..

[35] Swalwell H., Kirby D.M., Blakely E.L., Mitchell A., Salemi R., Sugiana C., Compton A.G., Tucker E.J., Ke B.X., Lamont P.J. (2011). Respiratory chain complex I deficiency caused by mitochondrial DNA mutations. Eur. J. Hum. Genet..

[36] Boczonadi V., Ricci G., Horvath R. (2018). Mitochondrial DNA transcription and translation: Clinical syndromes. Essays Biochem..

[37] Ghezzi D., Zeviani M. (2018). Human diseases associated with defects in assembly of OXPHOS complexes. Essays Biochem..

[38] Catteruccia M., Verrigni D., Martinelli D., Torraco A., Agovino T., Bonafe L., D'Amico A., Donati M.A., Adorisio R., Santorelli F.M. (2014). Persistent pulmonary arterial hypertension in the newborn (PPHN): A frequent manifestation of TMEM70 defective patients. Mol. Genet. Metab..

[39] Brito S., Thompson K., Campistol J., Colomer J., Hardy S.A., He L., Fernandez-Marmiesse A., Palacios L., Jou C., Jimenez-Mallebrera C. (2015). Long-term survival in a child with severe encephalopathy, multiple respiratory chain deficiency and GFM1 mutations. Front. Genet..

[40] Salviati L., Sacconi S., Murer L., Zacchello G., Franceschini L., Laverda A.M., Basso G., Quinzii C., Angelini C., Hirano M. (2005). Infantile encephalomyopathy and nephropathy with CoQ10 deficiency: A CoQ10-responsive condition. Neurology.

[41] Montini G., Malaventura C., Salviati L. (2008). Early coenzyme Q10 supplementation in primary coenzyme Q10 deficiency. N. Engl. J. Med..

[42] Vantroys E., Larson A., Friederich M., Knight K., Swanson M.A., Powell C.A., Smet J., Vergult S., De Paepe B., Seneca S. (2017). New insights into the phenotype of FARS2 deficiency. Mol. Genet. Metab..

[43] Friederich M.W., Timal S., Powell C.A., Dallabona C., Kurolap A., Palacios-Zambrano S., Bratkovic D., Derks T.G.J., Bick D., Bouman K. (2018). Pathogenic variants in glutamyl-tRNA(Gln) amidotransferase subunits cause a lethal mitochondrial cardiomyopathy disorder. Nat. Commun..

[44] Formosa L.E., Mimaki M., Frazier A.E., McKenzie M., Stait T.L., Thorburn D.R., Stroud D.A., Ryan M.T. (2015). Characterization of mitochondrial FOXRED1 in the assembly of respiratory chain complex I. Hum. Mol. Genet..

[45] Galmiche L., Serre V., Beinat M., Zossou R., Assouline Z., Lebre A.S., Chretien F., Shenhav R., Zeharia A., Saada A. (2012). Toward genotype phenotype correlations in GFM1 mutations. Mitochondrion.

[46] Pagani F., Baralle F.E. (2004). Genomic variants in exons and introns: Identifying the splicing spoilers. Nat. Rev. Genet..

[47] Perez B., Vilageliu L., Grinberg D., Desviat L.R. (2014). Antisense mediated splicing modulation for inherited metabolic diseases: Challenges for delivery. Nucleic Acid Ther..

[48] Holmes-Hampton G.P., Crooks D.R., Haller R.G., Guo S., Freier S.M., Monia B.P., Rouault T.A. (2016). Use of antisense oligonucleotides to correct the splicing error in ISCU myopathy patient cell lines. Hum. Mol. Genet..

