# Supplementary material for: Genes and Variants Underlying Human Congenital Lactic Acidosis—From Genetics to Personalized Treatment
Source: J Clin Med. 2019 Nov 1;8(11):1811. doi: 10.3390/jcm8111811 (PMC6912785; doi:10.3390/jcm8111811)
Supplement: Supplementary file 1 [file jcm-08-01811-s001.pdf]

Genes and variants underlying congenital lactic acidosis: from genetics to personalized treatments

Supplementary figures

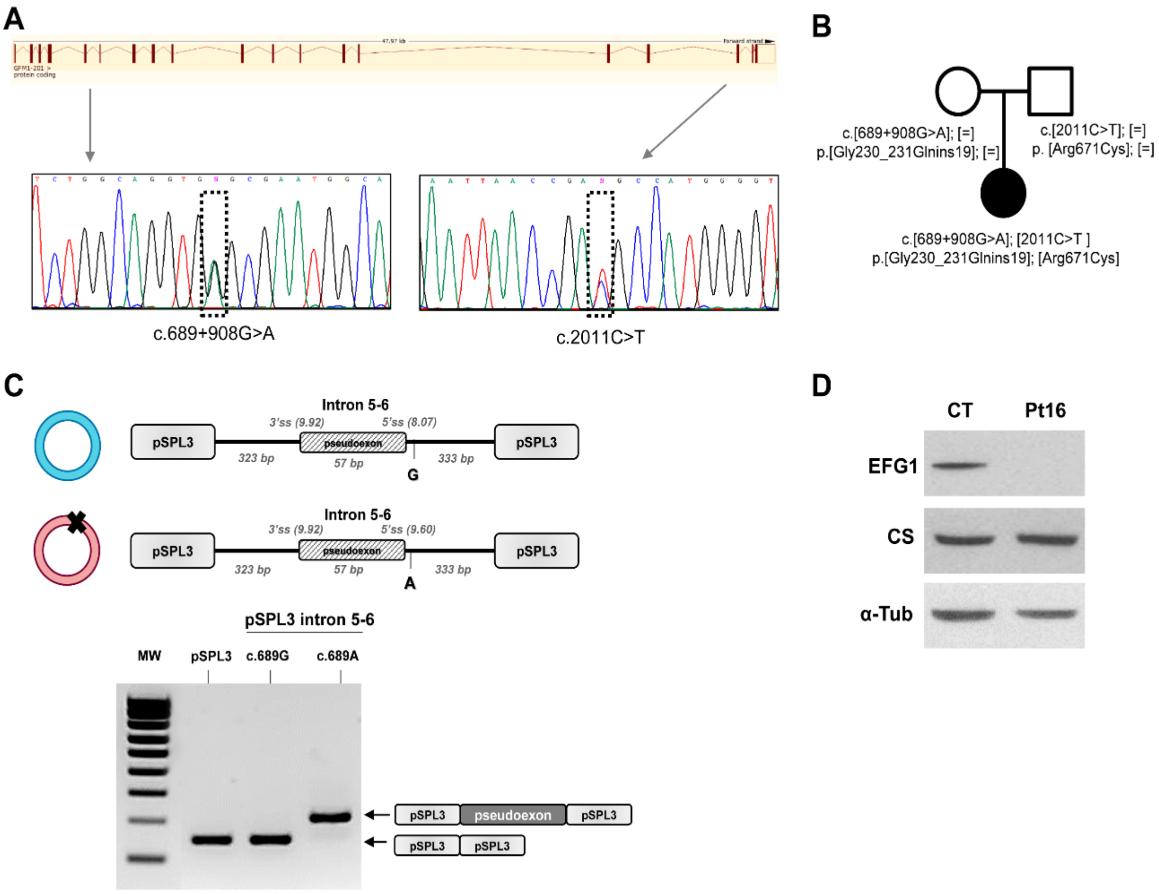

Figure S1. Genomic characterization of variant c.689+908G>A and effect of the change on minigene-splicing profile. A) Structure of the GFM1 gene and detail of the Sanger-sequenced region around c.689+908G>A and c.2011C>T. On the right of the figure is the family pedigree. B) Minigene analysis showing a diagram of the normal and mutant pSPL3 constructs, and gel electrophoresis of the RT-PCR products for the tested minigene. The adjacent diagram is of the variant-induced change in transcript configuration. The identity of the bands was confirmed by sequence analysis.

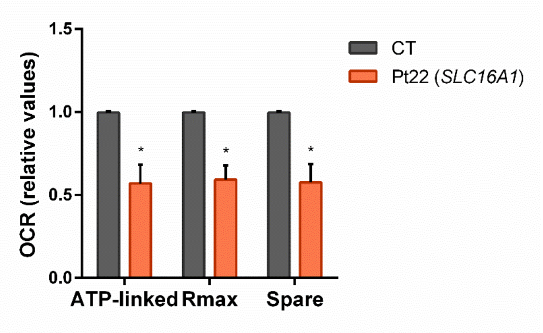

Figure S2. Oxygen consumption rates of fibroblasts from Pt22. The data shown are for ATP-production-dependent, maximal respiration (Rmax) and spare capacity (Spare). Results are expressed as fold over the control concentrations and are the mean ± SD of 3-5 wells from n=2-3 independent experiments. Control values are the means of two different control cell lines. Student t test (*p<0.05).

Supplementary information

Supplementary Tables

Table S1. Clinical phenotypes.

Pt Code/ Gender	Family history /Prenatal data	Age of onset	Age of death	Neurological and muscular features	Multisystem features	Imaging	Biochemical and laboratory findings
**Pt1.1/ F (a) Pt1.2/ M (b)**	Bi-amniotic twins; Foetal suffering	Neonatal onset HP:0003623	36 h; 3m		Cardiomyopathy HP:0001638; Neonatal respiratory distress HP:0002643	Delayed myelination HP:0012448	Metabolic acidosis HP:0001942
**Pt2/ M**	No consanguinity; Premature birth HP:0001622 (35wk); Decreased foetal movements HP:0001558	Neonatal onset HP:0003623 (12h)	11d		Hypotension HP:0002615; Patent ductus arteriosus HP:0001643; Optic disc pallor HP:0000543 (papillary pallor); Cutis laxa HP:0000973		Metabolic acidosis HP:0001942; Abnormal levels of creatine kinase in blood HP:0040081; Hypoglycaemia HP:0001943
**Pt3/ F**		Infantile onset HP:0003593 (3m)	Alive at 6y	Global developmental delay HP:0001263; Hypotonia HP:0001264; Spastic diplegia HP:0001264	Cardiomyopathy HP:0001638; Nephrotic syndrome HP:0000100; Cataract HP:0000518; Failure to thrive HP:0001508; Dysmorphic facies HP:0001999	Cerebral atrophy HP:0002059; Leukoencephalopathy HP:0002352	Metabolic acidosis HP:0001942
**Pt4/ M**	No consanguinity; Premature birth HP:0001622 (31wks); Severe intrauterine growth retardation HP:0008846; Oligohydramnios HP:0001562	Neonatal onset HP:0003623 (from birth); Congenital onset HP:0003577	Alive at 3y6m	Global developmental delay HP: 0001263	Grade II preterm intraventricular haemorrhage HP:0030749	Cerebellar hypoplasia HP:0001321; Enlarged foetal cisterna magna HP:0011427	Metabolic ketoacidosis HP:0005979
**Pt5/ F**	No consanguinity; Abnormal delivery HP:0001787 (foetal suffering)	Neonatal onset HP:0003623 (12h)	9d	Central hypotonia HP: 0011398; Myoclonic encephalopathy HP:0010851; EEG with burst-suppression HP:0003198; Myopathy HP:0003198	HP: 0001399 Hepatic failure		Metabolic acidosis HP:0001942; Abnormal levels of creatine kinase in blood HP:0040081; Elevated hepatic transaminase HP:0002910; Hypoglycaemia HP:0001943
**Pt6/ M**	No consanguinity Intrauterine brain	Neonatal onset HP:0003623 (7d)	¿?	Generalized hypotonia HP:0001290; Cortical dysplasia HP:0002539	Microcephaly HP:0000252; Growth delay HP:0001510	Agenesis of corpus callosum HP:0001274; Abnormality of neuronal migration	Metabolic acidosis HP:0001942

Supplementary information

Pt Code/ Gender	Family history /Prenatal data	Age of onset	Age of death	Neurological and muscular features	Multisystem features	Imaging	Biochemical and laboratory findings
	malformation (Brain atrophy HP:0012444)					HP:0002269; Holoprosencephaly HP:0001360	
**Pt7/ F**		Neonatal onset HP:0003623 (1d)	35d	Infantile axial hypotonia HP:0009062; Hypertonia HP:0001276; Limb dysmetria HP:0002406	Cholestasis HP:0001396		Metabolic acidosis HP:0001942; Hypoglycaemia HP:0001943
**Pt8/F**	Term	Infantile onset HP:0003593 (11m)	6y	Infantile muscular hypotonia HP:0008947; Global developmental delay HP: 0001263	Severe sensorineural hearing impairment HP:0008625; Growth delay HP: 0001510	Normal	Metabolic acidosis HP:0001942; Hypoglycaemia HP:0001943
**Pt9/ F**	Foetal suffering	Neonatal onset HP:0003623 (from birth)	3d	Infantile axial hypotonia HP:0009062; Not suction; Neonatal respiratory distress HP:0002643	Hepatic steatosis HP: 0001397	Partial agenesis of the corpus callosum HP:0001338; Abnormality of the cerebral white matter HP:0002500; Ventriculomegaly HP:0002119	Metabolic acidosis HP:0001942; Elevated hepatic iron concentration HP:0012465; Anaemia HP:0001903; Hypoglycaemia HP:0001943
**Pt10/ M**	No consanguinity; Bi-amniotic twins; Premature birth HP:0001622 (34wk); Intrauterine growth retardation HP:0001511	Infantile onset HP:0003593 (7m)	Died at 29m	Generalized hypotonia HP:0001290; Global developmental delay HP: 0001263	Pulmonary arterial hypertension HP:0002092; Failure to thrive HP:0001508;	Abnormality of the cerebral white matter HP:0002500;	Metabolic acidosis HP:0001942;
**Pt11/ F**	Oligohydramnios HP:0001562	Neonatal onset HP:0003623 (from birth)	Alive at 3y	Psychomotor retardation HP:0025356; Irritability HP:0000737; Excessive daytime sleepiness; somnolence HP:0001262; Peripheral neuropathy HP:0009830; Global developmental delay; HP: 0001263	Renal Fanconi syndrome HP:0001994; Polycystic kidney dysplasia HP:0000113; Growth delay HP:0001510	Abnormality of the basal ganglia HP:0002134; Leigh syndrome; Bilateral necrosis of Grey nuclei	Secondary hyperparathyroidism HP:0000867; Proteinuria HP:0000093; Hyperphosphaturia HP: 0003109
**Pt12/ M**	No consanguinity; Premature birth HP:0001622; Polyhydramnios HP:0001561	Childhood onset HP:0011463 (20m)	Alive at 3.5y	Central hypotonia HP:0011398; Seizures HP:0001250; Motor delay HP:0001270; Hypsarrhythmia	Bilateral cochlear hearing loss; Dolichocephalic	Hyper intensities lesions in Globus pallidus	Normal

Supplementary information

Pt Code/ Gender	Family history /Prenatal data	Age of onset	Age of death	Neurological and muscular features	Multisystem features	Imaging	Biochemical and laboratory findings
				HP:0002521; Hypertonia HP:0001276			
**Pt13/ M**	No consanguinity; Intrauterine growth retardation HP:0001511; Oligohydramnios HP:0001562	Neonatal onsetHP:0003623 (2d)	5d	Generalized hypotonia HP:0001290; EEG with burst suppression HP:0010851	Corneal opacity HP:0007957; Dysmorphic facies HP:0001999		Metabolic acidosis HP:0001942; Anaemia HP:0001903
**Pt14/ M**	No consanguinity	Infantile onset HP:0003593 (6m)	6m	Generalized hypotonia HP:0001290; Excessive daytime somnolence HP:0001262; Irritability HP:0000737; Mitochondrial encephalopathy HP:0006789	Arterial hypertension HP:0000822; Bradycardia HP:0001662; Mydriasis HP:0011499	Cerebral oedema HP:0002181; Increased intracranial pressure HP:0002516	Thrombocytopenia
**Pt15/ M**	No consanguinity; Abnormal delivery HP:0001787 (foetal suffering)	Neonatal onset HP:0003623 (from birth)	5y	Global developmental delay HP:0001263 Generalized hypotonia HP:0001290; Seizures HP:0001250; pyramidal-Abnormality of extrapyramidal motor function HP:0002071; EEG with abnormally slow frequencies HP:0011203	Intraventricular haemorrhage HP:0030746		Metabolic acidosis HP:0001942
**Pt16/ F**		Infantile onset HP:0003593 (9m)	Alive at 15m	Infantile axial hypotonia HP:0009062; Encephalopathy HP:0001298	Strabismus HP:0000486	Hypoplasia of the corpus callosum HP:0002079; Leukoencephalopathy HP:0002352 Delayed myelination HP:0012448	
**Pt17/ F**		Infantile onset HP:0003593 (2.5m)	Alive at 11y	Infantile axial hypotonia HP:0009062; Irritability HP:0000737; Encephalopathy HP:0001298; Epileptic spasms HP:0011097; Global developmental delay HP: 0001263		Cerebral atrophy HP:0002059 Frontal cortical atrophy HP:0006913 Temporal cortical atrophy HP:0007112Subcortical cerebral atrophy HP:0012157; Delayed myelination HP:0012448;	Metabolic acidosis HP:0001942 compensated Elevated hepatic transaminase HP:0002910; Increased mean platelet volume HP:0011877

Supplementary information

**Pt Code/ Gender**	**Family history /Prenatal data**	**Age of onset**	**Age of death**	**Neurological and muscular features**	**Multisystem features**	**Imaging**	**Biochemical and laboratory findings**
						Hypoplasia of the corpus callosum HP:0002079	
**Pt18/ M**	Term	Neonatal onset HP:0003623 (from birth)	Alive at 6y	Normal psychomotor developmental			Metabolic acidosis HP:0001942; Hyperamonemia HP:0001987; Elevated hepatic transaminase HP:0002910; Hypoketotic hypoglycaemia HP:0001985
**Pt19/ M**		Infantile onset HP:0003593 (4m)	Alive at 10m	Psychomotor deterioration HP:0002361; Epileptic encephalopathy HP:0200134; Epileptic spasms HP:0011097 (West syndrome)	Abnormal visual fixation HP:0025404; Microcephaly HP:0000252	Dysplasia occipital; Hypoplasia of the brainstem HP:0002365	
**Pt20/ F**		Infantile onset HP:0003593 (3m)	Alive at 1y	Central hypotonia HP:0011398; Hypertonia HP:0001276; Encephalopathy HP:0001298; Arrhythmia HP:0011675	Abnormal visual fixation HP:0025404	Bilateral alteration of central nucleus and of Corpus callosum	
**Pt21/ F**	No consanguinity	Neonatal onset HP:0003623 (from birth)	Alive at 9m	Mild global developmental delay HP:0011342		at 4 months; slight cerebral cortical atrophy HP:0002120 in MRI	Metabolic acidosis HP:0001942
**Pt22/ F**	Consanguinity	Infantile onset HP:0003593 (6m)	2y3m	Mild global developmental delay HP:0011342		Agenesis of Corpus callosum HP:0001274; lesions in brainstem	Metabolic acidosis HP:0001942; Ketosis HP:0001946
**Pt23/ M**	No consanguinity; Died cousin	Neonatal onset HP:0003623 (24d)	¿?	Irritability HP:0000737	Dilated cardiomyopathy HP:0001644; Horizontal nystagmus HP:0000666	Normal	Abnormality of coagulation HP:0001928
**Pt24/ F**	Consanguinity	Infantile onset HP:0003593 (9m)	Alive 2y	Psychomotor deterioration HP0002361; Infantile axial hypotonia HP:0009062; Abnormal pyramidal sign HP:0007256	Blindness HP:0000618	Leukodystrophy HP:0002415; cerebral hypomyelination HP:0006808	Metabolic acidosis HP:0001942; Ketonuria; Anaemia HP:0001903
**Pt25/ F**	No consanguinity; Preterm; Hydrops foetalis HP:0001789	Neonatal onset HP:0003623 (from birth)	1d		Cardiomyopathy HP:0001638; Abnormality of the kidney	Lissencephaly HP:0001339	Metabolic acidosis HP:0001942; Elevated hepatic transaminase HP:0002910

Supplementary information

Pt Code/ Gender	Family history /Prenatal data	Age of onset	Age of death	Neurological and muscular features	Multisystem features	Imaging	Biochemical and laboratory findings
					HP:0000077; hepatomegaly HP: 0002240		
**Pt26/ F**	Consanguinity	Infantile onset HP:0003593 (2m)	7m	Psychomotor retardation HP:0025356; seizures HP:0001250; Laryngomalacia HP:0001601	Nystagmus HP:0000639; Horseshoe kidney HP:0000085 Dysmorphic facies HP:0001999	Agenesis of corpus callosum HP:0001274	
**Pt27/ F**		Neonatal onset HP:0003623 (5d)	24d	Seizures HP:0001250; Coma HP:0001259	Renal insufficiency HP:0000083; Acute hepatic failure HP:0006554	Massive cerebral oedema HP:0002181	Metabolic acidosis HP:0001942; Hyperammonemia HP:0001987; Elevated hepatic transaminase HP:000291; Hyperbilirubinemia HP:0002904; Hypertriglyceridemia HP:0002155 Hyperglycaemia HP:0003074 (diabetic mother); Abnormality of the coagulation cascade HP:0003256
**Pt28/ M**	No consanguinity; Abnormal delivery HP:0001787 (foetal suffering)	Neonatal onset HP:0003623 (1d)	10d		Renal insufficiency HP:0000083	Abnormality of the basal ganglia HP:0002134; Ischemic lesions in the basal ganglia (basal ganglia necrosis HP:0012128); Diffuse cerebral oedema HP:0002181	Metabolic acidosis HP:0001942; Thrombocytopenia HP:0001873; Abnormality of the coagulation cascade HP:0003256
**Pt29/ M**		Infantile onset HP:0003593 (3m)	Alive 1.5y	Psychomotor retardation HP:0025356; Abnormal cry HP:0025429; Epileptic spasms HP:0011097 (West syndrome)	Visual fixation instability HP:0025405; Failure to thrive HP:0001508; Vomiting HP:0002013		
**Pt30/ F**	No consanguinity	Infantile onset HP:0003593 (6m)		Generalized hypotonia HP:0001290; Epileptic spasms HP:0011097 (West syndrome)			Metabolic acidosis HP:0001942

Supplementary information

Pt Code/ Gender	Family history /Prenatal data	Age of onset	Age of death	Neurological and muscular features	Multisystem features	Imaging	Biochemical and laboratory findings
**Pt31/ M**	Premature birth HP:0001622 (32 wks); Hydrops foetalis HP:0001789 (twins)	Neonatal onset HP:0003623 (from birth)	Alive 1y	Epileptic spasms HP:0011097 (West syndrome); Developmental regression HP:0002376	Grade I preterm intraventricular haemorrhage HP:0030748; Bronchopulmonary dysplasia ORPHA:70589 (abnormal respiratory system morphology HP:0012252; Abnormal lung morphology HP:0002088; Respiratory distress HP:0002098	Abnormality of the cerebral white matter HP:0002500	
**Pt32/ F**	No consanguinity; Premature birth HP:0001622 (36wks); Intrauterine growth retardation HP:0001511	Neonatal onset HP:0003623 (8d)	Alive at 6m	Good neuromuscular tone	Hypertrophic cardiomyopathy HP:0001639; Tachypnea HP:0002789; Bradycardia HP:0001662; Hepatomegaly HP:0002240; Feeding refusal (poor appetite HP:0004396)		Metabolic acidosis HP:0001942; Anaemia HP:0001903
**Pt33/ M**	No consanguinity	Neonatal onset HP:0003623 (1d)	Alive at 23y		Renal Fanconi syndrome HP:0001994; Cholestasis HP:0001396; Failure to thrive HP:0001508		Metabolic acidosis HP:0001942; Elevated hepatic transaminase HP:0002910; proteinuria HP:0000093; Hyperphosphaturia HP: 0003109
**Pt34/ M**		5m	Alive at 13m		Cardiomyopathy HP:0001638		Metabolic acidosis HP:0001942
**Pt35/ M**	Consanguinity; Term	Neonatal onset HP:0003623 (11d)	Alive at 2y2m		Hepatomegaly HP:0002240; Hepatic steatosis HP:0001397; Hepatic failure HP:0001399; Malnutrition HP:0004395	Normal	Elevated hepatic transaminase HP:0002910; Hyperbilirubinemia HP:0002904; Hyperammonemia HP:0001987;
**Pt36/ M**	Previous died brother	Infantile onset HP:0003593 (1m)	Alive at 36m	Global developmental delay HP:0001263; Irritability HP:0000737; Acute encephalopathy HP:0006846; Epileptic spasms		Abnormality of the cerebral white matter HP:0002500	Metabolic acidosis HP:0001942

Supplementary information

Pt Code/ Gender	Family history /Prenatal data	Age of onset	Age of death	Neurological and muscular features	Multisystem features	Imaging	Biochemical and laboratory findings
				HP:0011097 (West syndrome)			
**Pt37/ F**	Term	Infantile onset HP:0003593 (1.5m)		Generalized hypotonia HP:0001290; Encephalopathy HP:0001298; Generalized myoclonic seizures HP:0002123	Recurrent urinary tract infections HP:0000010; Abnormal involuntary eye movements HP:0012547; Abnormal facial shape HP:0001999		
**Pt38/ F**		Neonatal onset HP:0003623 (from birth)	Alive at 4m	Infantile axial hypotonia HP:0009062	Feeding refusal (Poor appetite) HP:0004396)		Hypoglycaemia HP:0001943
**Pt39/ F**	Term	Neonatal onset HP:0003623 (28d)	Alive at 3y10m	Neurologic hyper excitability		Leigh syndrome	Metabolic acidosis HP:0001942

Abbreviations: Female (F); male (M); hours (h); days (d); weeks (wk); months (m); years (y). HPO terms follow Human Phenotype Ontology terms (https://hpo.jax.org/app/); a and b biamniotic twins (only a was considered).

Table S2. Biochemical data.

Pt Code	Lactate plasma	Lactate CSF	Others plasma	Acyl-Carnit plasma	Lactate urine	Organic Acids urine	Cell studies	MDC
**Pt1.1 (a)** **Pt1.2 (b)**	12 7.4		Ala 2X; Tau 4.3X Ala: Normal	Normal	10396	2HB; 4HPL; 4HPP	Mitochondrial proliferation in M; Moderately decreased RC in M (a)and FB (b)	Definite
**Pt2**	16.7		Ala: Normal	C0	23167	2HB; 3HB; 4HPL; 2HVA	CoQ10 Normal in FB	Definite
**Pt3**	12.2	3.9	Ala 1.4X; Cit 3.5X↓; Pipecolic↑	C0, C3, C4, C5OH, C10OH, C18OH	414	2HB; 3HB; AcAc; 3MGC	PC, PDH, 1-14C pyr; 2-14C pyr; 1-14C glutamate oxidation Normal in FB; RC Normal in M	Definite
**Pt4**	12.2		Ala 7X	C2; C3	127476	2HB; 3HB; AcAc; 4HPL; 4HPP; Fum; Mal; 3MGA; 3HGA; 3MGC; 3Hprop; 3HIVA; Adipic; 3H-DC10; orotic		Definite

Supplementary information

Pt Code	Lactate plasma	Lactate CSF	Others plasma	Acyl-Carnit plasma	Lactate urine	Organic Acids urine	Cell studies	MDC
**Pt5**	30		Ala 3.8 X; Tyr 3.1X		34000	2HB; 3HB	PC Normal in FB, RC and PDH Normal in M. No mtDNA depletion	Definite
**Pt6**	13.9	10.3	Ala 1.8X		1444	2HB; 3HB; AcAc; 4HPA; Fum; Mal	PC Normal in FB	Definite
**Pt7**	20		No data		10854	2HB; 3HB; AcAc; 4HPA; 4HPL; 4HPP; 3Hprop; 2HIVA; 3H-DC10:1	PC Normal in FB	Definite
**Pt8**	9		Ala 1.7X	Normal	1368	2HB; 3HB; AcAc; 2-KGA; Fum; 3Hprop; 3H-DC10:1	PC Normal in FB	Definite
**Pt9**	11.7	13.2	Aa: Normal		5528	2HB; 4HPL; 3HIB; 2HIVA; Fum	RC Normal in liver, CII+III slightly decreased in FB; PC Normal in FB	Definite
**Pt10**	7.1		Aa: Normal	C2	Normal	Succ; Fum; Mal; 3MGC; 2HGA; 3H-3MGA	RC slightly decreased in FB; CoQ10 decreased in LF	Definite
**Pt11**	5.5		No data	C5-DC, C6-DC	4946	2HB; 3HB; 4HPL; 4HPP; AcAc; 2-KGA; 2HGA; GA; Fum; 2H-Adip, 2K-Adip,	↓CI, CIII and CIV in M; Myopathic alteration with signs of mitochondrial alteration	Definite
**Pt12**	4.7	2.87	Ala 2.1X	Normal	337	Normal	RC Normal in M	Definite
**Pt13**	20		Ala 2.7X; Lys 2.5X; Pro 1.9X; Tyr 3.8X; Cystathionine 6.8X	Normal	26399	2HB; 4HPL; 4HPP; 3HIB; Fum; 3MGA; 3MGC	PC Normal in FB	Definite
**Pt14**	4.1	5.8	Aa: Normal	Normal	681	2HB; 3HB; AcAc; Vanilmandelic;	PC Normal in FB	Definite
**Pt15**	4.5-5.03	3.93	Aa: Normal		Normal	Normal	PC, PDH, 1-14C pyr, 2-14C pyr; 1-14C glutamate oxidation Normal in FB and RC Normal in M	Probable
**Pt16**	4	2.19	Ala: Normal	Normal	Normal	Normal		Probable
**Pt17**	5.2	7.8	Ala 1.7X				PC Normal in LF	Probable
**Pt18**	13.8	7.5	Val 2.4X; Ile 2X; Leu 3.6X; Tyr 2.X; Lys 2.6X; Cit 4.4X; AloIleu ↑	C16:1OH	204258	2HB; 4HPL; 4HPP; AcAc; 2-KGA; 2HIVA; 2H,3M-VA; 2H-isocaproic; 2HGA; 3Hprop	Dihydrolipoamide dehydrogenase activity <3% in FB	Probable

Supplementary information

Pt Code	Lactate plasma	Lactate CSF	Others plasma	Acyl-Carnit plasma	Lactate urine	Organic Acids urine	Cell studies	MDC
**Pt19**	3.1-10.6		Ala: Normal	Normal	200	Normal		Probable
**Pt20**	6	8	Cit 2.4X↓	C0	2827	2HB; 3HB; 4HPP; 2-KG; Fum	PC and Dihydrolipoamide dehydrogenase activity Normal in FB; RC Normal in M	Probable
**Pt21**	14.7		Ala 4.6X; Pro 3.3X; Cit not detectable		1917	2HB; 3HB, AcAc, 3Hprop, 2M,3HB, 3HIVA, Fum, Mal, 2H-Glut	PC Normal and PDH Decrease in FB	Probable
**Pt22**	7	1.7	Aa: Normal values; Ketone Bodies↑	Normal	9312	2HB; 3HB; AcAc; 3Hprop; 2HIVA; 3HIVA		Probable
**Pt23**	6.4	1.77	Ala 2X; Pro 2.4X; Met 2.7X; Tyr 2.9X; uric acid 1.5X	↓C0, C3	7274	2HB; 3HB; 4HPP; 4HPL; AcAc; 2-KGA; Fum	PC; PDH; 1-14C pyr; 2-14C pyr; 1-14C glutamate oxidation in FB Normal	Probable
**Pt24**	11.7		Ala 3X	C6, C8:1, C8, C10:1, C10	4151	2HB; 3HB; AcAc; 2-KG; Succ, Fum; Mal; 4HPP; 2HGA, 3HGA, 3HIVA, 3Hprop	PC Normal in LF, PDH and RC Normal in M	Probable
**Pt25**	11.5		no data	Normal	2772	2HB; 2-KG; Succ; Fum; Mal; 3Hprop, GA, 2HGA, 4HPL	PC Normal in FB	Probable
**Pt26**	3	3.81	Aa: Normal	Normal	1958	2HGA, 2-KGA, Succ, Fum, 4HPA	PC; PDH; 1-14C pyr; 2-14C pyr; 1-14C glutamate oxidation in FB Normal, RC Normal in M	Probable
**Pt27**	16.6		Pro 1.8X	C0; C2-C6	63607	2HB; 3HB; AcAc; 4HPP; 4HPL; Homovanilic	PC Normal in FB; RC Normal in M	Probable
**Pt28**	9.1	9.42	Aa: Normal	C0			PC 43% in FB	Probable
**Pt29**	6.1		Ala 1.3X		Normal	Normal	RC Normal in M; no mtDNA depletion; PC 39% in FB	Probable
**Pt30**	8	2.82	Ala 2.1X		4497	2HB; AcAc; 3Hprop; 3HIVA; 4HPA; Valproate treatment		Probable
**Pt31**	4.0-3.0		Ala 2.6X	Normal	5869	2HB; 3HB; AcAc; 3Hprop; 3HIVA; Valproate treatment	Slightly decrease of CoQ10 in LF	Probable

Supplementary information

Pt Code	Lactate plasma	Lactate CSF	Others plasma	Acyl-Carnit plasma	Lactate urine	Organic Acids urine	Cell studies	MDC
**Pt32**	11	Normal	no data		66425	2HB; 3HB; AcAc; 4HPL; 4HPP; 3Hprop; 2HIVA	PC Normal in LF and FB; PDH Normal FB	Probable
**Pt33**	Elevated		Cit 2X		Elevated	KB	CIII deficiency in M	Possible
**Pt34**							PC Normal in FB	Possible
**Pt35**	11.6		Cit 2X; Val, Ileu+Leu N		3340	2HB; 4HPL; 4HPP; 2HIVA; 3HIVA; 2H-Adipic; 2K-Adipic	Dihydrolipoamide dehydrogenase activity <10% in FB	Possible
**Pt36**	4.0-6.0		Cit 2X	Normal	Normal	2H-Adipic	PC and PDH Normal in FB	Possible
**Pt37**	5.6		Ala: Normal	Normal		2-KGA		Possible
**Pt38**	18		Ala 1.6X; Cit 7.4X↓	Normal	328807	2HB; 3HB; AcAc; 4HPL; Fum; Mal; 3Hprop; 2HIVA; 2M, 2HB; 3HIVA; 3H-DC10:1	PC Normal in LF; RC Normal in M	Possible
**Pt39**	↑↑		Aa: Normal; Free thiamine in whole blood not detectable	Normal	1010	2-KGA		

Abbreviations: Amino acids (Aa); Ketone Bodies (KB); AcAc (acetoacetic); hydroxy (H); methyl (M); iso (I); keto (K); Valeric (VA); butyric (B) dicarboxylic acids (Adipic and Suberic) (DC); 4-OHphenyllactic (4HPL); 4-OHphenylpyruvic (4HPP); 4-OHphenylacetic (4HPA); Fumarate (Fum); Malate (Mal); Succinate (Succ); Glutaric (GA); Glutaconic (GC); Lactic (L); Pyruvic (P); Propionic (prop); Ethylmalonic (EMA)); Increased presence of C0: free carnitine, C2: acetylcarnitine, C3: propionylcarnitine, C4: butyryl+isobutyrylcarnitine, C5DC: glutarylcarnitine, C5OH: 3-hydroxyisovalerylcarnitine, C6: hexanoylcarnitine, C6DC: adipyl+methylglutarylcarnitine, C8: octanoylcarnitine, C8:1: octenoylcarnitine, C10: decanoylcarnitine, C10:1: decenoylcarnitine, C10OH: 3-hydroxydecanoylcarnitine, C16:1: palmitoleylcarnitine, C16:1OH: 3-hydroxypalmitoleylcarnitine, C18OH: 3-hydroxyestearoylcarnitine. [a)]Thiamine responsive lactic acidosis; Fibroblasts (FB); Muscle (M); Lymphocytes (LF); Pyruvate carboxylase (PC); Pyruvate dehydrogenase (PDH); Respiratory chain (RC); Coenzyme Q10 (COQ10). Normal lactate value in blood 1.8±0.5mmol/L; in CSF 1.6±0.3 mmol/L, and in urine 5-113 mmol/mol creatinine; Biochemical data for MDC scores: Lactate 2X in blood or CSF was scored as +2; alanine 2X was scored as +2; TCA (succinate, fumarate, malate); dicarboxylic acids (DC), 2HB, 3MGC were all scored as +1.

Table S3. Primers used for massive parallel sequencing of *GFM1* cDNA amplicons.

Primer	Tag Sequence	Target-Specific Sequence	Oligonucleotide Sequence	Tag Name	Target Name
**Forward Primer**	ACACTGACGACATGGTTCTACA	TTTCATCCGAGCAGGAGAAG	ACACTGACGACATGGTTCTACATTTCATCCGAGCAGGAGAAG	CS1	*GF*M1

**Reverse Primer**	TACGGTAGCAGAGACTTGGTCT	GCATGACCTAAGTTCAGTGGAA	TACGGTAGCAGAGACTTGGTCTGCATGACCTAAGTTCAGTGGAA	CS2	*GFM1*

Table S4. Prediction of pathogenicity by different tools.

Gene	Nucleotide Variation	Polyphen2 (HumVar)	Sift	Mutation Taster	PROVEAN	Mutation Assessor	Mutpred2 (score)	Panther (PSEP)
ACAD9	NM_014049.4: c.473C>T p.(Thr158Ile)	benign 0.36	DAMAGING score 0	polymorph	Deleterious - 4.48	High 3.75	Probably of deleterious mutation P= 0.780 (Loss of catalytic residue at T158 (P = 0.0286)	Not scored: No PANTHER family for input sequence
DLD	NM_000108.4: c.259C>T p.(Pro87Ser)	probably damaging 0.977	DAMAGING score 0	disease causing	Deleterious - 7.62	High 4.235	Probably of deleterious mutation P= 0.867	Not scored: No PANTHER family for input sequence
DLD	NM_000108.4: c.788G>A (p.Arg263His)	probably damaging 0.953	DAMAGING score 0	disease causing	Deleterious - 3.921	Medium 2.865	Probably of deleterious mutation P= 0.529	Not scored: No PANTHER family for input sequence
FARS2	NM_006567.3: c.737C>T p.(Thr246Met)	probably damaging 0.913	DAMAGING score 0.02	polymorph	Deleterious - 3.551	Medium 2.83	Probably of deleterious mutation P= 0.596	PROBABLY DAMAGING (842)
FARS2	NM_006567.3: c.1082C>T p.(Pro361Leu)	probably damaging 0.992	DAMAGING score 0.01	disease causing (0.99)	Deleterious - 9.192	High 3.54	Probably of deleterious mutation P= 0.907	PROBABLY DAMAGING (1629)
FOXRED1	NM_017547.3: c.628T>G p.(Tyr210Asp)	probably damaging 0.924	DAMAGING score 0.02	disease causing	Deleterious - 6.447	High 3.64	Probably of deleterious mutation P=0.796 Gain of disorder (p = 0.0129) Loss of sheet (p = 0.0315) Loss of phosphorylation at Y210 (p = 0.038)	Not scored: No PANTHER family for input sequence
FOXRED1	NM_017547.3: c.1273C>T p.(His425Tyr)	probably damaging 1.000	DAMAGING score 0	disease causing	Deleterious - 5.637	High 3.615	Probably of deleterious mutation P=0.650	Not scored: No PANTHER family for input sequence
GFM1	NM_024996.5: c.2011C>T p.(Arg671Cys)	probably damaging 1.000	DAMAGING score 0	disease causing	Deleterious - 7.06	High 4.165	Probability of deleterious mutation P=0.858	PROBABLY DAMAGING (4200)
MRPS22	NM_020191.2: c.509G>A p.(Arg170His)	probably damaging 1.000	DAMAGING score 0	disease causing	Deleterious - 4.40	Medium 3.19	Probability of deleterious mutation P=0.96	PROBABLY DAMAGING (1237)
NPHS2	NM_014625.3:c.413 G>A p.(Arg138Gln)	probably damaging 0.999	DAMAGING score 0.02	disease causing (0.99)	Deleterious - 3.839	Medium 2.47	Probably of deleterious mutation P= 0.821	PROBABLY DAMAGING (1237)

Supplementary information

Gene	Nucleotide Variation	Polyphen2 (HumVar)	Sift	Mutation Taster	PROVEAN	Mutation Assessor	Mutpred2 (score)	Panther (PSEP)
PDHA1	NM_000284.3: c.506C>T p.(Ala169Val)	probably damaging 0.985	DAMAGING score 0	disease causing (0.99)	Deleterious - 3.853	High 4.615	Probability of deleterious mutation P=0.950	PROBABLY DAMAGING (1629)
PDHA1	NM_000284.3: c.787C>G p.(Arg263Gly)	probably damaging 1.000	DAMAGING score 0	disease causing (0.99)	Deleterious - 6.542	High 3.95	Probably of deleterious mutation P= 0.927	PROBABLY DAMAGING (1237)
PDSS1	NM_014317.3:c.716 T>G p.(Val239Gly)	probably damaging 0.999	DAMAGING score 0	disease causing (0.99)	Deleterious - 6.456	Medium 3.345	Probability of deleterious mutation P=0.738 (Loss of stability (p = 0.0037)	Not scored: No PANTHER family for input sequence
PHKA2	NM_000292.2:c.124 6G>A p.Gly416Arg (ligada al X) EXAC browser 0.003668	probably damaging 0.975	DAMAGING score 0	disease causing (0.99)	Deleterious - 5.45	Medium 3.075	Probability of deleterious mutation P=0.312	PROBABLY DAMAGING (1036)
TRMU	NM_018006.4: c.680G>C p.(Arg227Thr)	probably damaging 0.982	DAMAGING score 0	disease causing (0.99)	Deleterious - 5.547	Medium 2.2 05	Probability of deleterious mutation P=0.708	Not scored: No PANTHER family for input sequence
TSFM	NM_001172696.1: c.782G>C p.(Cys261Ser)	Benign 0.02	DAMAGING score 0.02	disease causing (0.98)	Neutral -1.594	Low 1.21	Probability of deleterious mutation P=0.747 (Gain of disorder (p = 0.0016)	PROBABLY DAMAGING (455)
TSFM	NM_001172696.1: c.848G>A p.(Gly283Asp)	probable damaging 0.999	DAMAGING score 0	disease causing (0.99)	Deleterious - 6.592	Medium 2.925	Probability of deleterious mutation P=0.733 (Gain of relative solvent accessibility (p = 0.0215) Gain of solvent accessibility (p = 0.0306)	PROBABLY DAMAGING (1368)

In PolyPhen-2 (v2.2.2r398; http://genetics.bwh.harvard.edu/pph2/) using HumVar-trained model, scores above 0.9 were predicted as 'Probably Damaging', and those below 0.5 were predicted as 'benign". In SIFT (v1.03; http://sift.jcvi.org/), the cut-off for damaging prediction of 0.05. MutationTaster (http://www.mutationtaster.org/) employs a Bayes classifier, model simple aae (single amino acid changes) or complex aae (frameshift or premature stop codon). In PROVEAN the cut-off was 2.5. MutPred contains a general pathogenicity score (P) and a ranked list of specific molecular alterations potentially affecting the phenotype, where p is the P-value that certain structural and functional properties are impacted. Scores with $P > 0.5$ and $p < 0.05$ are actionable hypotheses; $P > 0.75$ and $p < 0.05$ are confident hypotheses, $P > 0.75$ and $p < 0.01$ are very confident hypotheses. For Panther analysis, PSEP (position-specific evolutionary preservation measured in length of time (millions of years). The thresholds chosen were: "probably damaging" (time > 450my; "possibly damaging" (450my > time > 200my) and "probably benign" (time < 200my).

Supplementary information
